# Mechanisms Linking Human Activities to Habitat Use by Amur Tigers and Amur Leopards Under Differentiated Management

**DOI:** 10.3390/ani16213480

**Published:** 2026-11-03

**Authors:** Haolun Fan, Zhikai Zheng, Xi Zheng

**Affiliations:** School of Landscape Architecture, Beijing Forestry University, 35 Qinghua East Road, Haidian District, Beijing 100083, China

**Keywords:** Amur tiger, Amur leopard, human disturbance, prey-mediated associations, seasonal space use, spatial-error SEM

## Abstract

Human pressures may be associated with predator space use directly or through prey, with variation by season and zoning context. We combined cameras, source-specific patrol observations, and environmental covariates across 18,243 km grids in Northeast China Tiger and Leopard National Park. Hierarchical models estimated predator spatial-use probabilities and prey use intensities using 2024 monthly inputs. Tigers used cameras and patrols, whereas leopard states used cameras only. Spatial-error piecewise SEMs used 500 independently paired state draws and 400 conditional multivariate coefficient simulations per pair. No direct path and two single-prey chains passed the extended descriptive screening rule during snow, compared with four direct paths and one chain in snow-free conditions. The four directional hypotheses received limited, pressure- and species-specific support; seasonal and B–H–M comparisons remained exploratory. Negative net indirect estimates sometimes arose from component signs contrary to the proposed resource-depletion pathway. Opposing farmland–leopard estimates produced an uncertain total rather than evidence of exact cancellation. Six park-wide alternatives examined estimation, covariate, and network assumptions; the original B–H–M models described four snow-free contexts. These conditional patterns support field assessment rather than causal mediation or intervention prescriptions.

## Simple Summary

We examined human-pressure, prey and predator spatial associations in Northeast China Tiger and Leopard National Park. Camera and source-specific patrol observations informed models of animal space use. Four directional working hypotheses and two exploratory comparisons distinguished prey–predator, pressure–prey, direct pressure–predator, and prey-mediated relationships. Adding approximate coefficient uncertainty reduced the number of screened pathways, especially during snow. Higher prey use did not consistently coincide with higher predator use. The retained B–H–M analysis describes four snow-free zoning contexts. These patterns guide field checks of pressure sources and monitoring coverage; they do not establish which interventions would improve predator recovery.

## 1. Introduction

Human disturbance can alter where large carnivores and their prey use a landscape. Roads, settlements, farmland, livestock activity, and illegal hunting may affect habitat access, prey resources, and predator risk through different pathways [1,2,3]. Fragmentation can separate suitable habitat patches, while disrupted corridors can restrict movement between them; prey depletion can further constrain resources available to recovering predators [4,5]. These processes motivate joint analysis of human activities, prey use, and predator space use.

Northeast China Tiger and Leopard National Park provides a shared landscape for examining these relationships. Existing research has described carnivore density, habitat and corridors, coexistence, prey selection, and activity patterns [6,7,8,9,10]. Human and livestock activities nevertheless occur within the park, including its core protection zones [3]. We therefore use current zoning as a management context alongside measured human pressures.

Two questions remain unresolved by these separate lines of evidence. A disturbance–predator association may arise directly within a conditional spatial model or indirectly through individual prey species. The same association may also vary by season and among areas combining different predator use, disturbance, and zoning. Seasonal studies establish changes in distributions and activity, but do not determine every spatial pathway within this park [11,12]. Comparing these sources requires separating ecological states from source-specific detection.

Our objectives are to estimate direct, prey-mediated, and total conditional associations between individual human-disturbance variables and predator spatial use; compare these associations between snow and snow-free seasons; and explore variation among estimable Biological value–Human disturbance–Management context (B–H–M) scenarios in the snow-free season.

We formulate four directional working hypotheses and two exploratory comparisons. The directional expectations follow evidence that prey distributions are associated with predator use and that human activities can affect both trophic levels [1,2,9,10,13,14].

**H1 (Prey–predator association).**  *Greater relative use intensity of sika deer, Siberian roe deer, and wild boar is positively associated with the spatial-use probability of Amur tigers and Amur leopards. Each prey–predator pathway is evaluated separately*.

**H2 (Human pressure–prey association).**  *Greater human pressure is hypothesized to be negatively associated with the relative use intensity of the three prey species. Pressure indicators comprise proximity to roads, farmland, and settlements, livestock activity, and snaring risk. The distance-derived predictors were coded so that larger values represent greater proximity pressure before standardization*.

**H3 (Direct human pressure–predator association).**  *After accounting for prey use, environmental covariates, and spatial error, greater human pressure is hypothesized to be negatively associated with tiger and leopard spatial-use probability. Here, direct denotes a model association that does not pass through the prey variables included in the specified network*.

**H4 (Prey-mediated association).**  *Greater human pressure is expected to be associated with lower predator use indirectly through reduced prey use. We evaluate prey-specific products, their net sum across prey, and the total association; a negative product supports this expectation only when its component signs follow the predicted pressure–prey and prey–predator sequence*.

**H5 (Seasonal comparison; exploratory).**  *The direct and prey-mediated associations for a given pressure–predator pairing may differ between the snow and snow-free seasons. We compare season-specific estimates and their uncertainty without predicting which season has the stronger association*.

**H6 (B–H–M scenario comparison; exploratory).**  *The associations among human pressure, prey use, and predator use may vary among estimable snow-free B–H–M scenarios. These scenarios combine predator-use level, human pressure, and institutional zoning; differences are interpreted as context-specific associations, not effects of a management intervention*.

## 2. Materials and Methods

### 2.1. Analytical Framework

The two threatened, sympatric felids provide a basis for comparing predator associations in the same landscape while retaining species-specific state and observation models. Regional dietary and prey-selection studies identify sika deer, Siberian roe deer, and wild boar as important wild ungulate prey [9,10]; these are the prey species assessed here.

The analysis has two linked stages. Hierarchical models first estimate predator spatial-use probabilities and prey use intensities from source-specific observations. The archived predator model integrates cameras and patrols for tigers, while leopard states use cameras only. Prey models link camera counts to use intensity and patrol detections to latent use. Spatial-error piecewise SEMs then estimate conditional associations and separate direct, selected-prey indirect, net indirect, and total quantities. B–H–M stratification provides exploratory snow-free-season comparisons.

**Figure 1:**
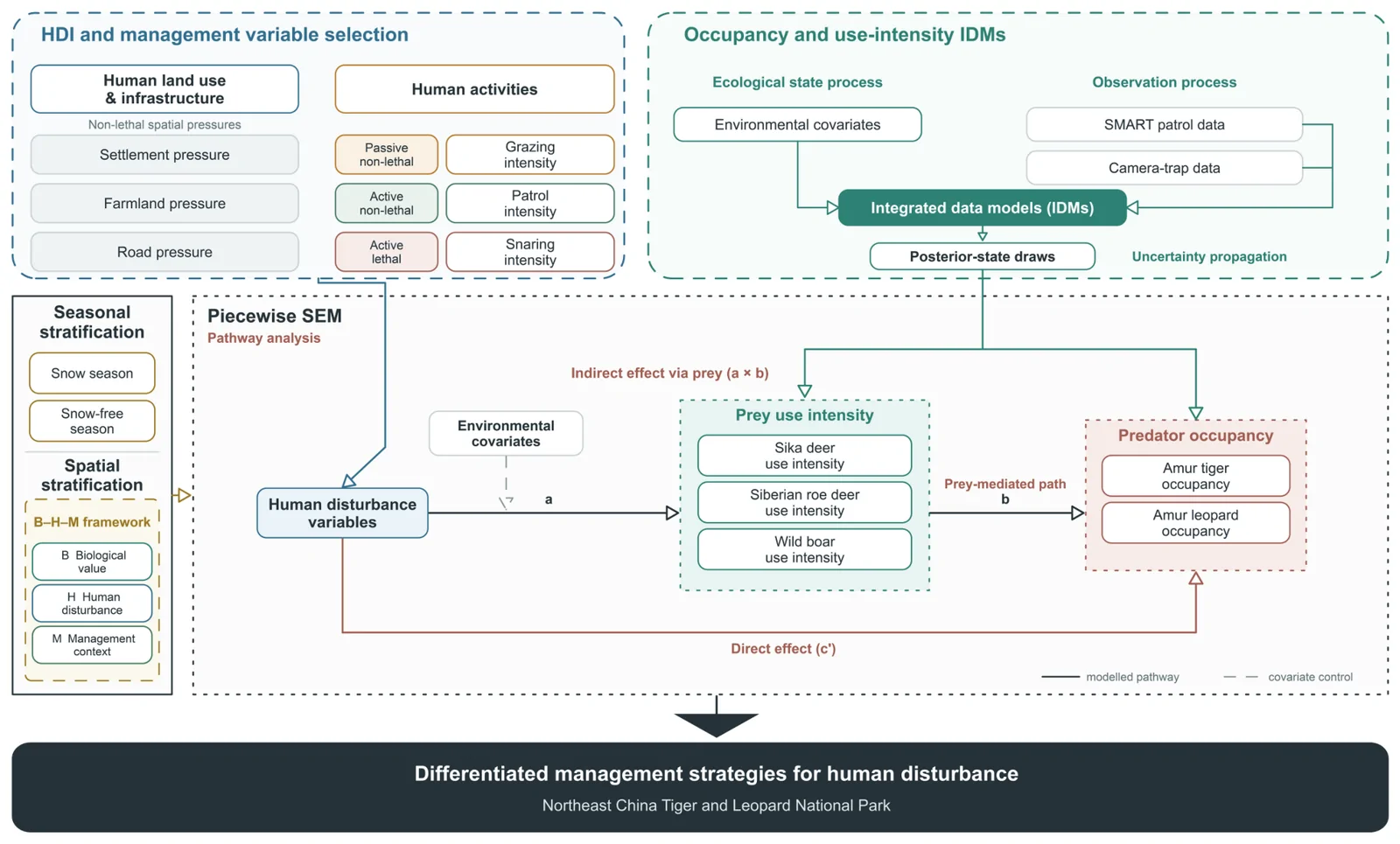
Framework linking human-pressure indicators, source-specific IDM estimates of predator spatial-use probability and prey use intensity, and seasonal spatial-error piecewise SEMs. Arrows denote modeled conditional pathways. Tiger estimates integrate camera and patrol observations; leopard estimates use cameras only. Patrol intensity is modeled separately from disturbance pressures. B–H–M denotes Biological value, Human disturbance, and Management context; scenarios guide field assessment.

The two-stage analytical workflow is summarized in Figure 1.

### 2.2. Study Area

**Figure 2:**
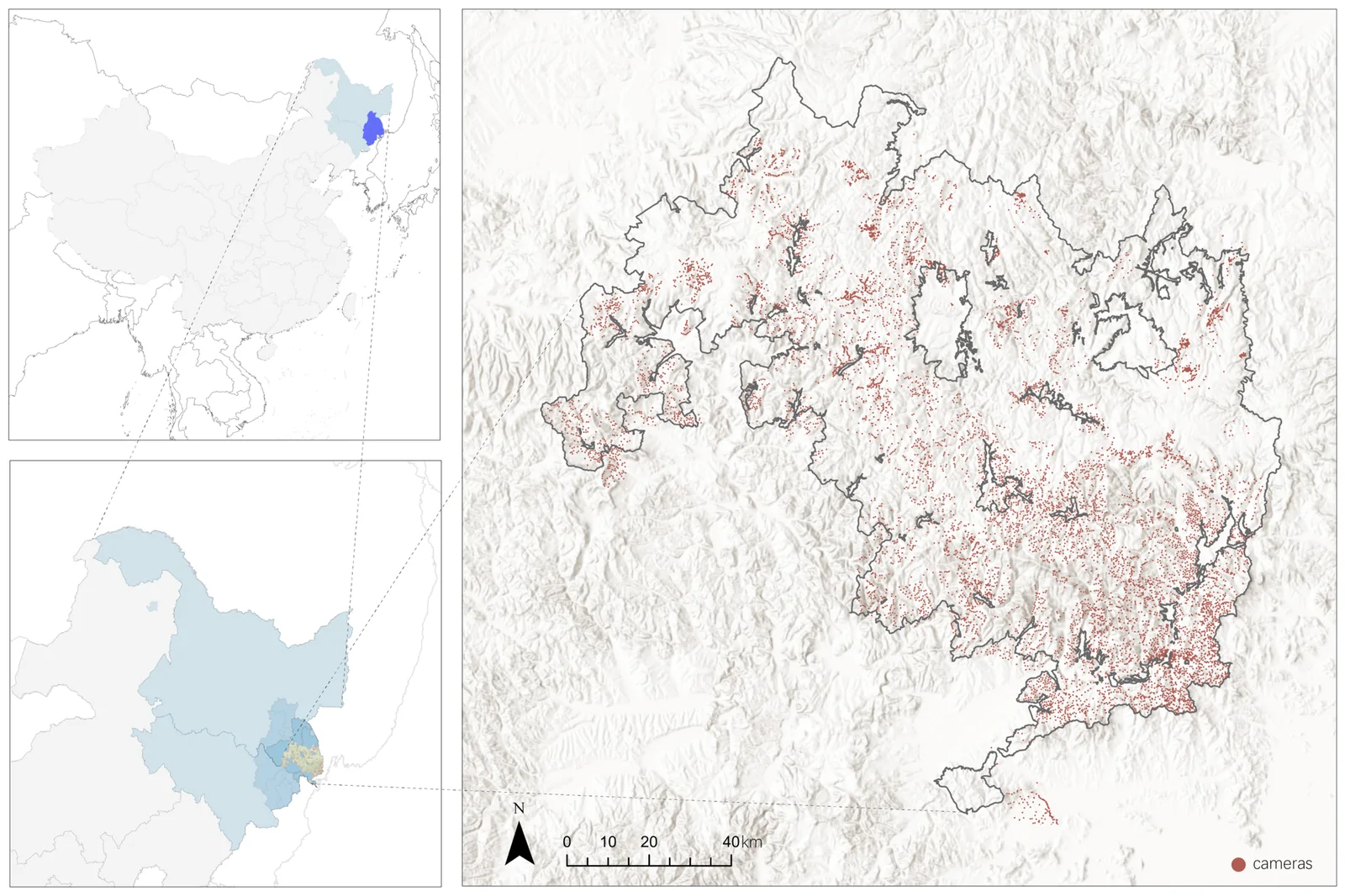
Study area, management zones, and monitoring-data distribution in Northeast China Tiger and Leopard National Park.

The park lies in northeastern China along the border between Jilin and Heilongjiang provinces. Its reported area is approximately 14,065 km^2^, including 9557 km^2^ in Jilin and 4508 km^2^ in Heilongjiang [15]. Temperate mixed coniferous–broadleaf and broadleaf forests dominate. Core protection and general control zones provide the institutional contexts used in the analysis (Figure 2). Roads, settlements, farmland, livestock activity, and patrol coverage vary within the landscape [2,15,16].

### 2.3. Data

#### 2.3.1. Data Sources

Table 1 summarizes the human disturbance, environmental, and animal-observation data. Disturbance variables described land use, infrastructure, and routine human activities. Following earlier work, we distinguished land-use and infrastructure pressures from activity-related pressures [1,17,18]. Camera-trap studies elsewhere show that direct human presence and fixed human footprints can affect mammals differently [19,20]; our classification applies that distinction to the available data. Distances to settlements, farmland, and roads represented land-use and infrastructure pressures. For SEMs, the distance-derived predictors were coded as proximity-pressure indicators before standardization, so larger values indicate greater proximity and stronger pressure; livestock, snaring, and patrol indicators were oriented so larger values indicate greater activity or effort. Activity-related variables included passive non-lethal disturbance (livestock activity), active non-lethal disturbance (patrol intensity), and lethal risk (snaring).

Environmental data described spatial variation in predator occupancy and prey use intensity, with initial variables selected from previous studies [21,22]. Animal observations provided the response data. Camera traps yield relatively continuous and standardized images, but each device samples a limited area and its effective detection field depends on placement [23]. Patrol records extend spatial coverage through sightings and sign detections, although detection varies with patrol routes and intensity, observer practices, ground conditions, and sign persistence [24]. Integrating both sources can produce more robust and spatially extensive estimates of latent wildlife use [23,25,26].

The archived livestock and snaring fields were completed with Gaussian identity-link GAMs fitted by REML, using environmental and human-pressure covariates, season, and a two-dimensional coordinate smooth (basis dimension 80). Observed values were retained. Livestock had 2280 observed and 1368 imputed entries among 3648 grid-seasons (37.5%); no snaring entries were imputed. On the training data, livestock RMSE was 0.268 and Spearman correlation was 0.556 on the archived scale. Completed covariates were standardized within season. A sensitivity analysis propagated GAM mean-function uncertainty to imputed livestock entries while retaining observed values and the original B–H–M classification (Supplementary Methods S1). The source files do not record the livestock variable’s original units or why 1368 values were unavailable, so completed values were treated as standardized relative-pressure indices.

#### 2.3.2. Monitoring Units and Sampling Coverage

Camera-trap and patrol data were provided by Northeast China Tiger and Leopard National Park.

Monitoring was reported for January 2024–January 2025. The archived predator V6 and prey V3 inputs contain 12 monthly periods, January–December 2024. January–March and December define the snow season; April–November define the snow-free season. January 2025 was not included in the fitted inputs, so the analyses cover the 2024 calendar year. These calendar-defined seasons do not imply uniform snow cover across grid-months.

Processed observations were organized by 3 km grid, species, season, source and month. Monthly aggregation aligned wildlife observations with monthly patrol-effort data and retained repeated observations within each season, the finest common temporal unit available in the supplied analytical records. Predator arrays include padded visits; missing entries were excluded from the source likelihood and recorded zeros were retained.

The park informed us that camera deployment and operation logs, including monthly active-camera counts, operating days, camera-days, and failure records, were unavailable. The supplied records also did not identify the interval used to define independent camera detections. We therefore could not reconstruct duration-based camera exposure or independently verify the event-independence interval. Grid-level camera-count proxies represent recorded differences in camera numbers but cannot account for unequal operating duration or camera failures.

Supplementary Table S1a–S1c reports source-specific observation coverage. Tiger cameras supplied 4560 valid grid-month inputs in snow and 9120 in snow-free conditions, including 210 and 279 positive grid-months. Tiger patrol inputs supplied 2438 and 2114 observations, including 10 and seven positive grid-months. Leopard cameras have the same visit counts, with 310 and 448 positive grid-months; leopard patrols did not inform the fitted likelihood. Prey camera-count sums were 23,199, 55,170, and 9903 in snow, and 130,161, 155,485, and 24,548 in snow-free conditions for sika deer, Siberian roe deer, and wild boar, respectively.

The IDMs separate ecological state processes from source-specific observation processes, so observed detections are not treated as the ecological quantities themselves. Predator states represent spatial-use probabilities, and prey states represent relative use intensities rather than animal abundance or density. Differences in camera numbers among grids were represented by grid-level camera-count proxies: prey models used counts as exposure, and predator models used fixed log-count effort terms. Monthly patrol effort entered the observation models where that source was included. Photographs, signs, source-specific visits, and positive grid-months have distinct units; input shares describe composition rather than statistical information.

#### 2.3.3. Data Processing

Data processing involved four main components: data-source processing, spatial-scale selection, environmental covariate selection, and variable standardization.

(1) Data-source processing.

As shown in Table 1, each data source was transformed according to its observation properties to meet IDM requirements. Multisource occupancy research emphasizes the need to represent source-specific detection and sampling processes [27]. We integrated multisource observations for multiple species within a Bayesian hierarchical IDM and converted them into grid-by-month observation matrices [26,28,29].

(2) Spatial-scale selection.

To determine an appropriate spatial scale for the analysis, we used a 500 m grid as the base spatial unit and aggregated these cells into 1 km, 3 km, and 5 km grids. Multisource occupancy models with the same model structure were then fitted independently at each spatial scale. Following previous studies [30,31,32], scale suitability was evaluated based on four criteria: spatial resolution, availability of detection information, model stability, and suitability for subsequent SEM analyses. The specific comparison metrics are presented in Table 2.

The 3 km grid provides a common spatial unit for aligning observations and covariates. It reduced sparse detections relative to 1 km cells while retaining more spatial detail than 5 km cells; the selected scale also balanced model stability and suitability for the SEM (Table 2). The grid is an analytical unit, not a shared home range for all five species.

(3) Environmental covariate selection.

To limit complexity in the IDM state process and reduce covariate redundancy, we first used Bayesian additive regression trees (BART) for species-specific screening of the environmental variables in Table 1 [33,34]. BART is a Bayesian nonparametric ensemble of regression trees that can identify nonlinear responses and relative variable importance [33]. Final state-process covariates were selected based on ecological relevance, collinearity diagnostics, and model interpretability [35].

(4) Variable standardization.

Continuous predictors in the IDM state process were standardized before model fitting. This facilitated comparison across measurement scales and improved numerical stability during Markov chain Monte Carlo (MCMC) fitting. Binary indicators and observation-effort offsets were not standardized in the same way as continuous predictors.

SEM prey states were transformed as Z[log(1+λ)] and predator states as $Z[\mathrm{logit}\left(\psi^{*}\right)]$, with $\psi^{*}$ truncated to [0.001,0.999]. Within each fitted draw and season, *Z* centers and scales by the sample standard deviation; exogenous variables were standardized in each fitted dataset. Coefficients describe standardized associations on transformed states, and scenario estimates also reflect the narrower variation within each scenario.

#### 2.3.4. Descriptive Comparisons

**Figure 3:**
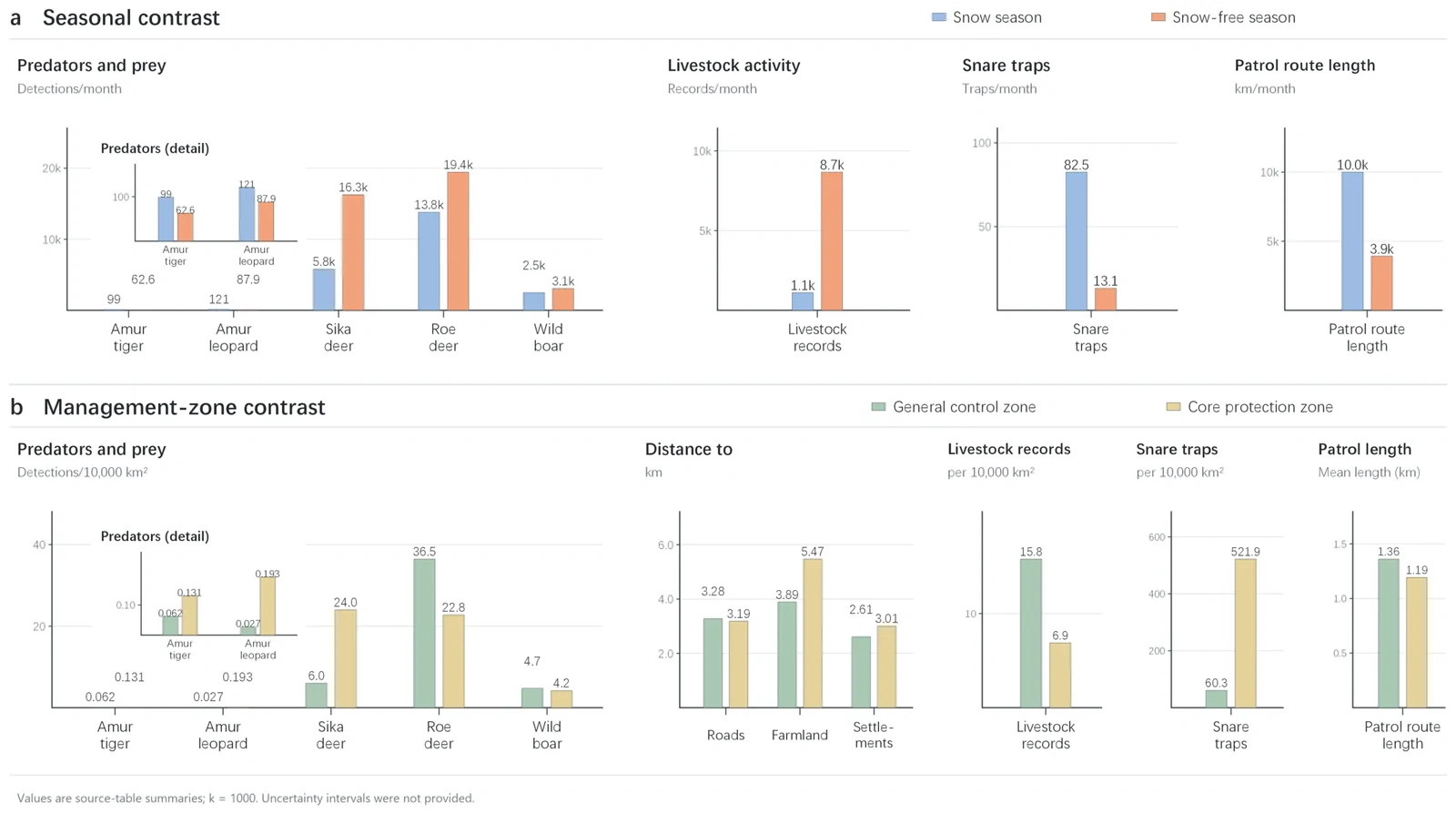
Seasonal and management-zone differences in major wildlife records, human activities, and patrol input. (**a**) Snow and snow-free seasons; (**b**) general control and core protection zones.

Before detection modeling, we summarized wildlife, livestock, and snaring records and patrol-route length by season and institutional zone (Figure 3). Seasonal values are monthly records; zoning summaries use area-based source units. These are descriptive contrasts rather than detection-corrected abundance or effort-standardized rates; distance indicators represent proximity to infrastructure.

**Table 2:** Evaluation metrics for spatial-scale suitability.

Metric Category	Evaluation Metric	Interpretation and Purpose
Spatial resolution	Number of valid grids ($N_{\mathrm{grid}}$)	Quantifies the number of spatial sampling units available for modeling at each scale and evaluates the balance between spatial resolution and sample size.
Detection information	Grid-level detection- positive rate	Evaluates whether sufficient detection information is available for the target species at each spatial scale and helps avoid unstable model estimation caused by too few positive grids.
Model stability	AUC	Evaluates discrimination between detection-positive and detection-negative grids. When calculated on the fitting data, AUC measures apparent discrimination and does not, by itself, establish spatial transferability [36,37].
	MCMC convergence diagnostic ($\hat{R}$)	Assesses parameter convergence at each spatial scale and identifies potential between-chain inconsistencies or model instability [38].
	Effective sample size ($n_{\mathrm{eff}}$ or ESS)	Evaluates the stability and effective information content of posterior estimates [38].
SEM suitability	Mean grid-level spatial-use probability ($\bar{\psi }$) and its spatial gradient	Evaluates whether model outputs have clear ecological interpretability and whether they are suitable as input variables for subsequent SEM analyses.

### 2.4. Analytical Methods

#### 2.4.1. Integrated Data Models

Integrated data models (IDMs) combine multiple data sources within a shared hierarchical model and joint likelihood [26,29]. In ecology, they are used to estimate species distributions, occupancy, abundance, and population trends while representing source-specific observation processes. The key distinction is between the latent ecological state and the observations generated from it.

This separation allows sources with different detection mechanisms to inform a common ecological state while accounting for observation error, imperfect detection, and variation in survey effort [25,26,29]. We constructed occupancy IDMs for the predators and occupancy–use-intensity IDMs for the prey within a Bayesian hierarchical framework. The models comprised an ecological state process, an observation process, and procedures for fitting, diagnosis, and uncertainty propagation.

(1) Ecological state process.

For these wide-ranging predators, ψ is the latent probability that a grid was used during a season, which we call spatial-use probability. It describes seasonal grid use rather than residency, abundance, or population density [25,28].

Let $z_{i,k,s}$ indicate whether predator species *k* potentially used grid *i* during season *s*. The ecological state process was specified as

(1) $$z_{i,k,s}∼\mathrm{Bernoulli}\left(\psi_{i,k,s}\right),$$

where $\psi_{i,k,s}$ denotes the latent spatial-use probability of predator species *k* in grid *i* during season *s*. The predator model jointly represented the snow and snow-free seasons as

(2) $$\begin{array}{cc} \mathrm{logit}(\psi_{i,k,s})= & \beta_{0,k}+\beta_{\mathrm{season},k}I_{\mathrm{snow},s}+\sum_{m=1}^{M_{k}}\beta_{\mathrm{state},m,k}X_{i,k,m}^{\mathrm{state}}\\ \; & +I_{\mathrm{snow},s}\sum_{q=1}^{Q_{k}}\beta_{\mathrm{int},q,k}X_{i,k,q}^{\mathrm{int}}+\beta_{\mathrm{snow},k}X_{i,k}^{\mathrm{snow}}I_{\mathrm{snow},s}. \end{array}$$

Here, $I_{\mathrm{snow},s}$ equals 1 for the snow season and 0 for the snow-free season. $X_{i,k,m}^{\mathrm{state}}$ denotes a species-specific covariate with a common effect across seasons, whereas $X_{i,k,q}^{\mathrm{int}}$ denotes a covariate whose additional snow-season effect was represented by an interaction with $I_{\mathrm{snow},s}$. The composite snow-pressure covariate $X_{i,k}^{\mathrm{snow}}$ also entered only during the snow season. The model therefore included a seasonal intercept contrast, common state effects, explicit snow-season interaction effects, and a snow-pressure effect.

For the three prey species, which had substantially more detections than the two predators, we constructed occupancy–use-intensity IDMs. Unlike the joint seasonal predator model, the prey models were fitted independently for the snow and snow-free seasons using the 2024 observations. Within each seasonal model, prey states were separated into an occupancy component indicating whether a grid was potentially used and a latent use-intensity component λ describing relative resource use within used grids [28,39,40].

Let s∈{snow,snow-free} index the independently fitted prey model. For prey species *k*, the two state components were specified as

(3) $$z_{i,k}^{\left(s\right)}∼\mathrm{Bernoulli}\left(\psi_{i,k}^{\left(s\right)}\right),$$

(4) $$\mathrm{logit}\left(\psi_{i,k}^{\left(s\right)}\right)=\beta_{0,k}^{\left(s\right)}+\sum_{m=1}^{M_{k,s}}\beta_{m,k}^{\left(s\right)}X_{i,k,m}^{\mathrm{occ},(s)},$$

and

(5) $$\log\left(\lambda_{i,k}^{\left(s\right)}\right)=\gamma_{0,k}^{\left(s\right)}+\sum_{q=1}^{Q_{k,s}}\gamma_{q,k}^{\left(s\right)}X_{i,k,q}^{\lambda ,(s)}.$$

Because the two seasonal prey models were fitted separately, they did not include a shared season main effect or a season-interaction term. The archived V3 model uses species-specific covariates and an intercept-only binary use component for roe deer (Supplementary Table S2). The composite snow-pressure covariate was considered only in the snow-season model. For downstream notation, $\psi_{i,k}^{\left(s\right)}$ and $\lambda_{i,k}^{\left(s\right)}$ are also written as $\psi_{i,k,s}$ and $\lambda_{i,k,s}$. The latter is not a direct estimate of prey abundance; it represents relative latent use intensity relative to the available camera-count exposure proxy and the observation model. Compared with raw camera-trap encounter rates or patrol-record frequencies, λ provides a model-based representation of spatial variation in prey use conditional on the specified observation model [40].

(2) Observation process.

The archived predator model uses binary camera observations for both species and binary patrol observations for tigers. Leopard patrol observations were set to missing and did not inform the likelihood. For included sources *d*,

(6) $$y_{i,k,s,v}^{d}∼\mathrm{Bernoulli}\left(z_{i,k,s}p_{i,k,s,v}^{d}\right).$$

Camera detection was specified as

(7) $$\mathrm{logit}\left(p_{i,k,s,v}^{\mathrm{cam}}\right)=\alpha_{0,k}^{\mathrm{cam}}+O_{i,s,v}^{\mathrm{cam}}+\alpha_{\mathrm{season},k}^{\mathrm{cam}}I_{\mathrm{snow},s},$$

and tiger patrol detection as

(8) $$\mathrm{logit}\left(p_{i,k,s,v}^{\mathrm{patrol}}\right)=\alpha_{0,k}^{\mathrm{patrol}}+O_{i,s,v}^{\mathrm{patrol}}+\alpha_{\mathrm{season},k}^{\mathrm{patrol}}I_{\mathrm{snow},s}.$$

The JAGS code fixes the effort-term coefficients to one rather than estimating slopes. The fitted-object scaling metadata specifies centered log transformations with scale equal to one:

(9) $$O^{\mathrm{cam}}=\log\left(1+C\right)-2.026538,\;O^{\mathrm{patrol}}=\log\left(1+L\right)-1.816995,$$

where *C* is the archived Camera_Count_2024 field and *L* is grid-month patrol length in km. These offsets are centered, not divided by their standard deviations.

Each independently fitted seasonal prey model links camera counts to intensity through

(10) $$y_{i,k,s,v}^{\mathrm{cam},\mathrm{count}}∼\mathrm{NegBin}\left(\mu_{i,k,s,v},r_{k}\right),\;\mu_{i,k,s,v}=z_{i,k,s}\lambda_{i,k,s}E_{i,s,v}^{\mathrm{cam}}+10^{-5}.$$

The offset is $\log(E^{\mathrm{cam}})$, where the archived field is grid-level camera count. Missing-exposure visits have missing observations; the numerical placeholder used for their offset does not turn them into observed zeros. The latent intensity is relative to modeled camera exposure, not animal abundance or density.

Prey patrol observations inform the binary latent-use component through

(11) $$\mathrm{logit}\left(p_{i,k,s,v}^{\mathrm{patrol}}\right)=\alpha_{0,k}^{\mathrm{patrol}}+\alpha_{\mathrm{eff},k}^{\mathrm{patrol}}F_{i,s,v},$$

where *F* is the standardized log-transformed patrol-effort covariate. This estimated prey slope differs from the predator fixed effort term. Separately fitted seasonal prey models have no shared detection-season coefficient. Routes, sign persistence, recording practices, and snow conditions can still affect detection [24].

(3) Model fitting, diagnostics, and uncertainty propagation.

We fitted all IDMs in a Bayesian hierarchical framework using Markov chain Monte Carlo (MCMC) sampling, which jointly represented ecological states, observation processes, and parameter uncertainty [28]. Models were implemented in JAGS through the jagsUI interface in R [41].

The predator IDM used three MCMC chains with 30,000 iterations per chain, a burn-in of 15,000 iterations, thinning of 10, and 3000 adaptation iterations, yielding 4500 retained posterior samples. The prey occupancy–use-intensity IDMs were fitted separately for the snow and snow-free seasons; each seasonal prey model used three chains with 40,000 iterations per chain, a burn-in of 20,000 iterations, thinning of 10, and 5000 adaptation iterations, yielding 6000 retained posterior samples per season.

For readability, normal priors are reported as Normal(mean,variance); the reciprocal variances were supplied as precisions in JAGS. In the predator model, state and detection intercepts were assigned $\mathrm{Normal}(0,3.16^{2})$ priors, whereas season effects, snow effects, state-process covariate effects, seasonal interaction terms, and detection-season effects were assigned $\mathrm{Normal}(0,2^{2})$ priors. In the prey models, occupancy, use-intensity, and patrol-detection intercepts were assigned $\mathrm{Normal}(0,3.16^{2})$ priors; occupancy covariate effects were assigned $\mathrm{Normal}(0,1^{2})$ priors; use-intensity covariate effects and patrol-effort detection effects were assigned $\mathrm{Normal}(0,2^{2})$ priors; and the negative-binomial dispersion parameter used a Uniform(0.1,100) prior.

Model convergence and sampling stability were evaluated using multiple MCMC chains, $\hat{R}$, effective sample size (ESS), posterior trace plots, and posterior parameter intervals [38]. We used $\hat{R}<1.01$ as the main target for formal model convergence, interpreted ESS together with trace-plot mixing and posterior intervals, and treated larger $\hat{R}$ or low ESS values in candidate models as screening-stage risk signals rather than as stand-alone ecological evidence.

The main analyses were conducted in R 4.5.2. IDMs were fitted in JAGS 4.3.2 through jagsUI 1.6.3 and rjags 4-17. Spatial-error SEMs were fitted using errorsarlm() in spatialreg 1.4-3, with spdep 1.4-2 and Matrix 1.7-5 as dependencies. The final predator maximum $\hat{R}$ was 1.003 and minimum effective sample size was 976. Across all archived prey-parameter summaries, snow and snow-free maximum $\hat{R}$ values were 1.007 and 1.005, with minimum effective sample sizes of 427 and 605, respectively. These convergence summaries do not establish observation-model adequacy.

The archived analysis materials include the IDM JAGS model files, fitted objects, parameter summaries, posterior-state draws, SEM R scripts, draw indices, and output manifests. Because the raw camera locations, patrol routes, and threatened-species spatial records are sensitive, the full code, de-identified data dictionary, and reproducible input files are available from the corresponding author subject to approval by the relevant data authority.

We retained the posterior distributions of the latent ecological states so that state-estimation uncertainty could be propagated into the subsequent SEM analyses [42].

For each species *k*, grid *i*, and season *s*, the model generated posterior samples of latent occupancy probability:

(12) $$\left\{\psi_{i,k,s}^{\left(1\right)},\psi_{i,k,s}^{\left(2\right)},\ldots ,\psi_{i,k,s}^{\left(D\right)}\right\},$$

where *D* denotes the total number of retained posterior draws and $\psi_{i,k,s}^{\left(d\right)}$ represents the occupancy probability at posterior draw *d*. The posterior mean was calculated as

(13) $${\bar{\psi }}_{i,k,s}=\frac{1}{D}\sum_{d=1}^{D}\psi_{i,k,s}^{\left(d\right)}.$$

For prey species, posterior samples and posterior means of latent use intensity $\lambda_{i,k,s}$ were extracted in the same manner. Posterior means were used for descriptive summaries and spatial visualization, and as the inputs to alternative model A2; the primary SEM used posterior-state draws. For the SEMs, we independently paired 500 predator and prey posterior-state draws from their separately fitted models and refitted each model. This pairing propagated uncertainty in IDM state estimates but did not constitute sampling from a common joint posterior distribution. Throughout, *i* indexes grids, *k* species, *s* seasons, and *d* posterior draws.

#### 2.4.2. B–H–M Management Scenarios and Pathway Heterogeneity

We developed a three-axis Biological value–Human disturbance–Management context (B–H–M) framework to describe spatial mismatches and pathway heterogeneity. The framework draws on systematic conservation planning, which considers conservation value, threats, and management feasibility together [43], and on human-footprint research that combines roads, agriculture, settlements, and human access into cumulative disturbance measures [17,18,44].

The Biological value axis (*B*) was the mean annual spatial-use probability of Amur tigers and Amur leopards, which jointly characterized grid-level conservation value. The Human disturbance axis (*H*) was the HDI, calculated as the mean of standardized road, farmland, and settlement pressures, livestock activity intensity, and snaring intensity.

The Management context axis (*M*) followed the existing national park zoning system: core protection zones were classified as high-*M* contexts and general control zones as low-*M* contexts. This axis represents the institutional context embodied by the zoning system, not actual patrol input, enforcement intensity, or realized management effectiveness [45,46,47].

For the continuous *B* and *H* axes, grids above the 75th percentile were classified as high and those below the 25th percentile as low. Grids between these cutoffs formed the background class. We crossed only the high and low extremes of both axes with the two *M* categories, producing 2×2×2=8 focal scenarios. A grid remained in the background class if either *B* or *H* lay between the 25th and 75th percentiles; the framework was therefore not a 3×3×2=18-class cross-tabulation.

We used these scenarios to assess whether human disturbance–prey resource–apex predator pathways differed among biological-value, disturbance, and management contexts.

#### 2.4.3. Piecewise Structural Equation Modeling and Path-Based Effect Decomposition

##### Piecewise SEM

Using the IDM estimates, we constructed spatial-error piecewise structural equation models (piecewise SEMs) to estimate direct conditional associations between human disturbance and predator space use and indirect associations transmitted through prey. A piecewise SEM represents a hypothesized path network as linked local regressions and assesses the overall structure with tests of directed separation and Fisher’s *C* [48,49,50,51].

We fitted separate park-wide models for the snow and snow-free seasons.

The main network contains six human variables H (road, farmland and settlement pressures, livestock activity, snaring, and patrol intensity), three prey states $P_{m}$, and two predator states $Y_{k}$. The control vector C contains elevation, mean DBH, edge distance, water distance, NDVI, and snow pressure. All six human variables and controls enter each prey equation; all three prey additionally enter each predator equation. No predator–predator arrow or correlated innovation enters the main model (Supplementary Figure S1):

(14) $$\begin{array}{cc} P_{m,i} & =a_{0m}+a_{m}^{T}H_{i}+g_{m}^{T}C_{i}+u_{m,i}, \end{array}$$

(15) $$\begin{array}{cc} Y_{k,i} & =c_{0k}+c_{k}^{T}H_{i}+\sum_{m=1}^{3}b_{mk}P_{m,i}+h_{k}^{T}C_{i}+u_{k,i}, \end{array}$$

(16) $$\begin{array}{cc} u_{j} & =\rho_{j}Wu_{j}+\epsilon_{j}. \end{array}$$

Spatial dependence was modeled in the equation errors, with a separate spatial coefficient for each equation, rather than as a response lag. Supplementary Table S7 lists candidate paths and the comparisons used for H1–H4. We evaluated H1–H4 from the corresponding prey–predator, pressure–prey, and direct pressure–predator coefficients, together with prey-specific products and the net sum across prey; patrol intensity was assessed separately as a management covariate. Tables S3 and S7 report all estimates, including paths opposite to the predicted direction. We interpret each indirect estimate alongside its component signs. H5 compares seasonal estimates and intervals descriptively because the seasonal models were fitted separately. H6 uses the archived snow-free B–H–M summaries and directional screen, which were not recalculated with the extended coefficient uncertainty. Intervals are nominal and not adjusted for multiplicity.

Predator responses were IDM-estimated spatial-use probabilities and prey variables were IDM-estimated use intensities. We independently paired 500 posterior-state draws from the separate models and refitted the SEM for each pair. For each fit, we drew 400 multivariate Normal coefficient vectors from the estimated within-equation covariance, yielding 200,000 conditional simulations per season. Coefficients were sampled independently across equations. The intervals are conditional on each fit’s spatial parameter, completed covariates, and specified graph; they do not include joint posterior or network uncertainty. Supplementary Methods S1 describes covariance reconstruction and validation, as well as the livestock-imputation sensitivity.

To account for spatial autocorrelation in the grid data, the primary model fitted all structural equations and d-separation regressions as spatial error models using spatialreg::errorsarlm(). These models incorporate unexplained spatial dependence into the residual structure, reducing potential bias in pathway estimates and inference [52].

The primary weights connect 3 km-grid centroids within 4300 m and use row standardization. The 1824-grid graph has 2–8 neighbors per grid, no isolates, and one connected component. Structural equations and independence-test regressions use the same spatial-error specification. The six alternatives below apply to park-wide models; the B–H–M analyses retain their original specification.

The main basis set has three prey-pair independence claims conditional on human variables and controls, and a tiger–leopard claim conditional on all prey and exogenous variables. Each uses a spatial-error regression and two-sided Normal coefficient test. For each fitted draw, Fisher’s $C=-2\sum_{r=1}^{4}\log\left(p_{r}\right)$ is evaluated against $\chi^{2}$ with eight degrees of freedom. Log-scale probabilities avoid underflow. Table S4 reports conditioning sets, coefficient summaries, statistics, and rejection fractions; full per-draw tests are supplied as source data. These tests assess conditional independence within each fitted model and are distinct from the directional screening summaries.

Moran’s *I* was calculated for the specified spatial weights and filtered innovations [53].

Six archived alternatives test selected modeling choices: A1 replaces spatial-error regressions with ordinary linear models; A2 uses posterior-mean states; A3 substitutes a composite HDI for six human variables; A4 omits natural controls; A5 adds seasonal tiger–leopard residual association; and A6 adds tiger state to the leopard equation. A2 provides point estimates without state-uncertainty propagation. A1–A4 vary estimation or covariate choices, while A5–A6 change network structure. Some alternatives change more than one feature, and state draws are not paired across every model family; we therefore use them as targeted sensitivity checks rather than isolated effects or a model ranking. Table S5d–g give their definitions, pairing structure, estimates, and diagnostics.

##### Path-Based Effect Decomposition

We decomposed associations between human disturbance and tiger or leopard space use into direct, prey-mediated indirect, and total path associations [54,55,56]. For consistency with the equations, we refer to these quantities below as direct, indirect, and total effects. They are conditional associations under the hypothesized path structure, not causal mediation effects.

For a human disturbance variable *X*, prey variable $M_{m}$, and predator response *Y*, the prey-specific indirect effect at posterior-state draw *d* was defined as the product of the human disturbance–prey pathway coefficient and the prey–predator pathway coefficient:

(17) $${\mathrm{Indirect}}_{X\to Y,m}^{\left(d\right)}=\beta_{X\to M_{m}}^{\left(d\right)}\times \beta_{M_{m}\to Y}^{\left(d\right)},$$

where $M_{m}$ represents sika deer, Siberian roe deer, or wild boar.

The total indirect effect transmitted through all three prey species was calculated as

(18) $${\mathrm{Indirect}}_{X\to Y}^{\left(d\right)}=\sum_{m=1}^{3}\beta_{X\to M_{m}}^{\left(d\right)}\times \beta_{M_{m}\to Y}^{\left(d\right)}.$$

The total effect was defined as the sum of the direct effect and total indirect effect:

(19) $${\mathrm{Total}}_{X\to Y}^{\left(d\right)}=\beta_{X\to Y}^{\left(d\right)}+{\mathrm{Indirect}}_{X\to Y}^{\left(d\right)}.$$

A selected-prey indirect effect differs from the net sum across all three prey. We report a mediated proportion only when direct, net indirect, and total estimates have concordant directions and the total estimate is separated from zero. The descriptive ratio of medians is

(20) $$\mathrm{Mediated}\;\mathrm{proportion}=\frac{\left|\mathrm{median}\left({\mathrm{Indirect}}_{X\to Y}\right)\right|}{\left|\mathrm{median}\left({\mathrm{Total}}_{X\to Y}\right)\right|}\times 100\%.$$

When the direct and indirect associations had opposite signs, the total association approached zero, or the absolute indirect association exceeded the absolute total association, we reported only the direct, indirect, and total quantities. No combination met the full concordance conditions under the extended uncertainty assessment, so no mediated proportion is reported in the revised Results.

Points summarize medians of the 500 conditional estimates; bars show the 2.5th–97.5th percentiles of the state-and-coefficient simulations. A path meets the descriptive support rule when at least 95% of simulations share a sign and its interval lies wholly on that side of zero. A supported single-prey chain additionally requires both component coefficients and their product to meet the rule. Counts use unrounded values and identical candidate networks across seasons.

## 3. Results

### 3.1. Spatiotemporal Differences in Human Activities and Wildlife Records

Seasonal contrasts differed between trophic groups (Figure 3a). Predator records were slightly higher during snow, whereas the three ungulates had more snow-free records. Livestock records were concentrated in snow-free conditions; snare/trap records and patrol-route length were higher during snow. These are record and activity contrasts; unequal effort and detection prevent interpreting them as direct abundance changes.

The management zones also differed (Figure 3b). Records per unit area of Amur tigers, Amur leopards, and sika deer were higher in the core protection zone. Siberian roe deer records were more frequent in the general control zone, whereas wild boar records differed little. Core protection areas were generally farther from farmland and settlements, but their distances to roads were similar to those in the general control zone. Grazing records occurred mainly in the general control zone, while snare/trap records were higher in the core protection zone. Patrol-route length was slightly higher in the general control zone.

These seasonal and zoning contrasts motivate separate conditional models without determining path directions.

### 3.2. IDM Selection and Latent Space-Use Patterns

#### 3.2.1. Spatial-Scale Selection, Covariate Screening, and Model Diagnostics

The 3 km grid was retained as a working scale balancing detection information, convergence, spatial resolution, and sites available for SEM (Table 3). Apparent AUC was 0.832, compared with 0.775 and 0.806 at 1 and 5 km. Fitting-data discrimination does not establish spatial transferability or scale robustness of pathways.

The em dash for mean spatial-use probability at 1 km indicates that this value is not reported because the candidate models at that scale did not achieve adequate convergence and sampling stability.

The maximum $\hat{R}=1.0229$ and minimum ESS = 118 for the 3km scale in Table 3 came from the candidate models used during spatial-scale screening, not from the final posterior-state outputs used in the SEMs. In the final formal 3km predator IDM, the maximum $\hat{R}$ was 1.0026 and the minimum ESS was 976. In the prey seasonal models, the maximum $\hat{R}$ was 1.0066 and the minimum ESS was 427 when grid-level latent states were included; for monitored core parameters, the maximum $\hat{R}$ was 1.0049 and the minimum ESS was 536. We therefore treated the 1.0229/118 values as borderline screening diagnostics indicating the need for formal long-chain fitting and posterior-state uncertainty propagation, whereas the pathway analyses were based on the final formal posterior-state draws.

At the 3km scale, elevation, distance to permanent water, mean diameter at breast height, and snow pressure remained important in BART models for several species. Environmental covariates for each species were selected according to predictive performance, variable stability, collinearity, and ecological interpretability (Table 4).

#### 3.2.2. Predator Spatial-Use Probabilities and Prey Use-Intensity Patterns

**Figure 4:**
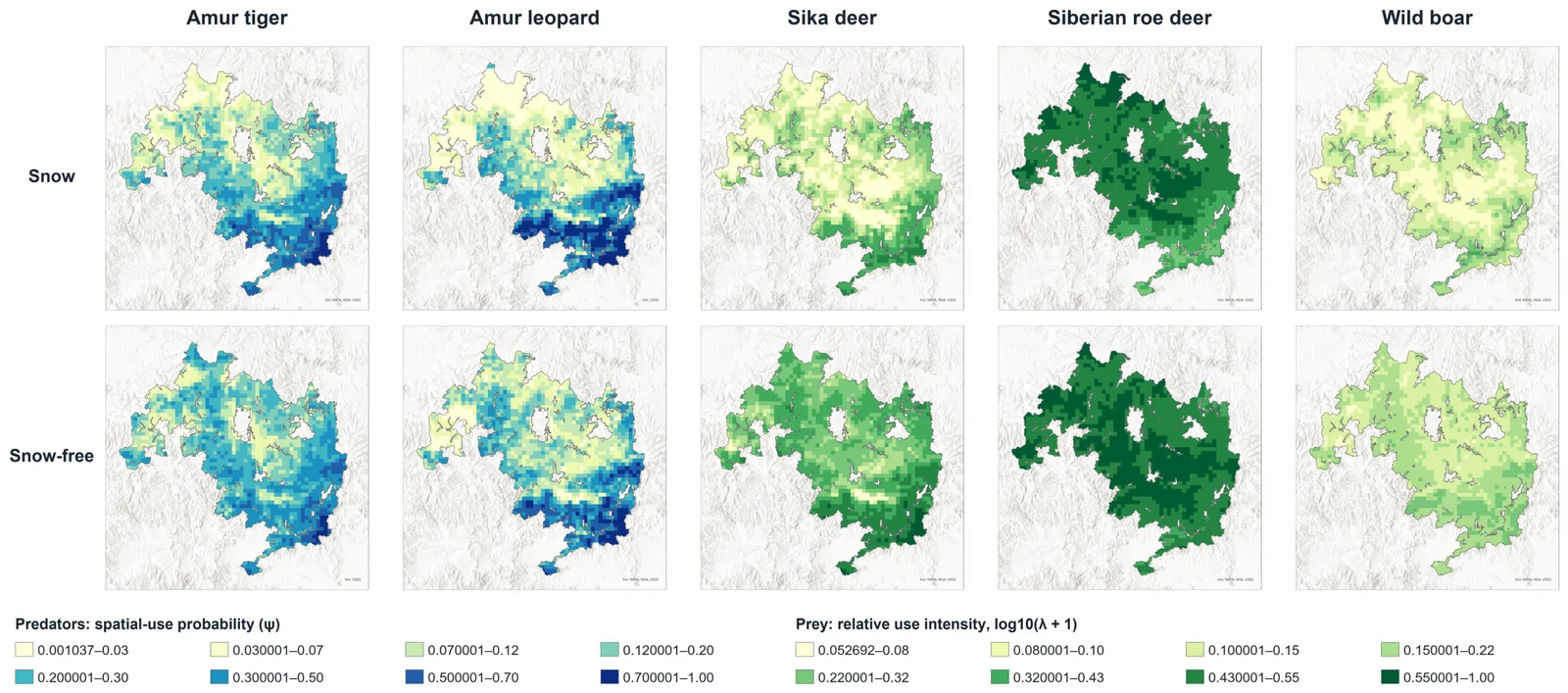
IDM-estimated space use of Amur tigers, Amur leopards, and their major prey. Predator maps show latent spatial-use probability ψ; prey maps show latent use intensity λ for sika deer, Siberian roe deer, and wild boar on a $\log_{10}\left(\lambda +1\right)$ scale.

IDM estimates showed marked spatial variation in latent use by both predators and all three prey species (Figure 4). Areas with high tiger and leopard spatial-use probabilities were concentrated in the south and southeast, with generally lower probabilities in the north and northwest. Predator high-probability areas were more continuous in the south and east during the snow-free season and contracted during the snow season.

The three prey species had distinct spatial patterns. Siberian roe deer had relatively high latent use intensity and the broadest distribution. Sika deer were concentrated in the south, southeast, and parts of the east, with a larger high-intensity area in the snow-free season. Wild boar use intensity was generally lower and more patchy in the snow season. Predator high-probability areas overlapped substantially with high-intensity areas of some major prey in the south and southeast.

### 3.3. Direct Human-Disturbance Associations and Prey-Mediated Pathways

#### 3.3.1. Seasonal Pathway Structure in the Park-Wide Spatial-Error Piecewise SEM

**Figure 5:**
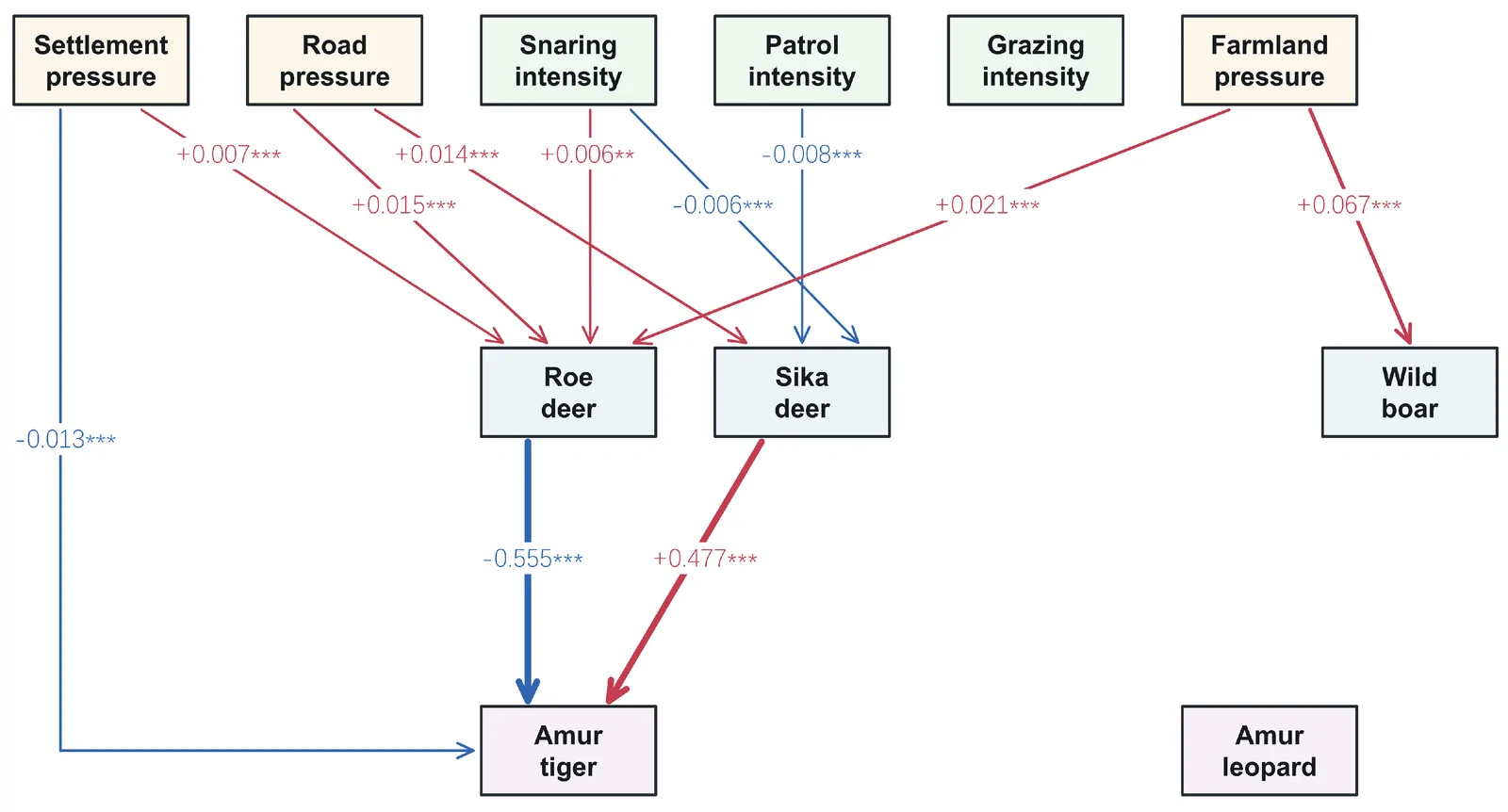
Submitted snow-season SEM diagram. Red and blue arrows indicate positive and negative associations, respectively. Two and three asterisks indicate that the direction was consistent in at least 97.5% and 99.5% of posterior-state draws, respectively. These markers summarize the original state-only screen and are not conventional *p*-value significance markers or current interval support. Updated estimates are in Figure 6.

**Figure 6:**
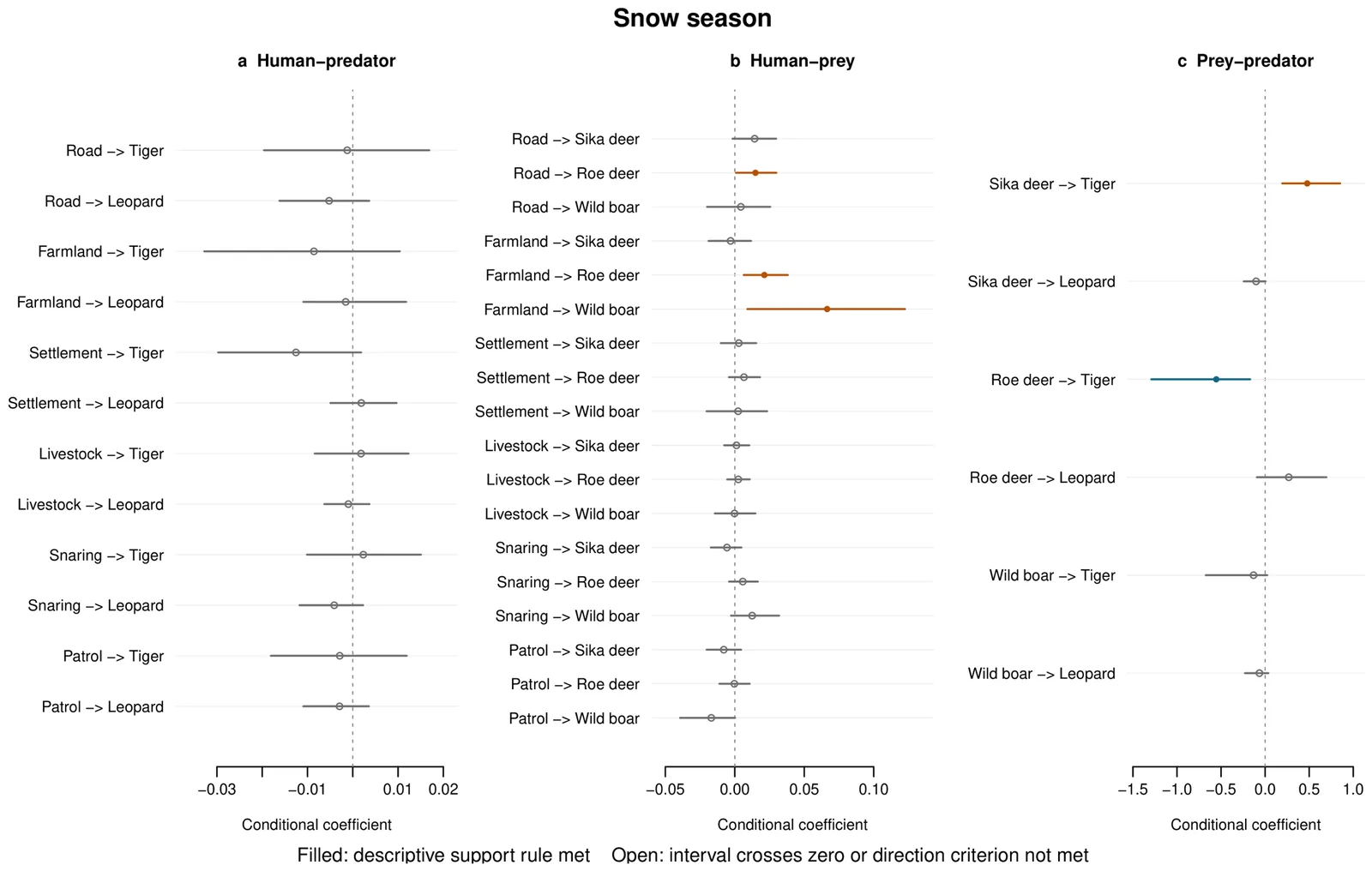
Park-wide conditional path estimates for the indicated season. Blue and orange points indicate negative and positive estimates, respectively. Points show medians of 500 state-conditioned estimates; bars show approximate 95% simulation intervals including state and within-equation coefficient uncertainty. Filled markers meet both descriptive screening criteria; open gray markers do not. Patrol intensity is a management covariate. Intervals are conditional on fitted spatial parameters and the specified network.

**Figure 7:**
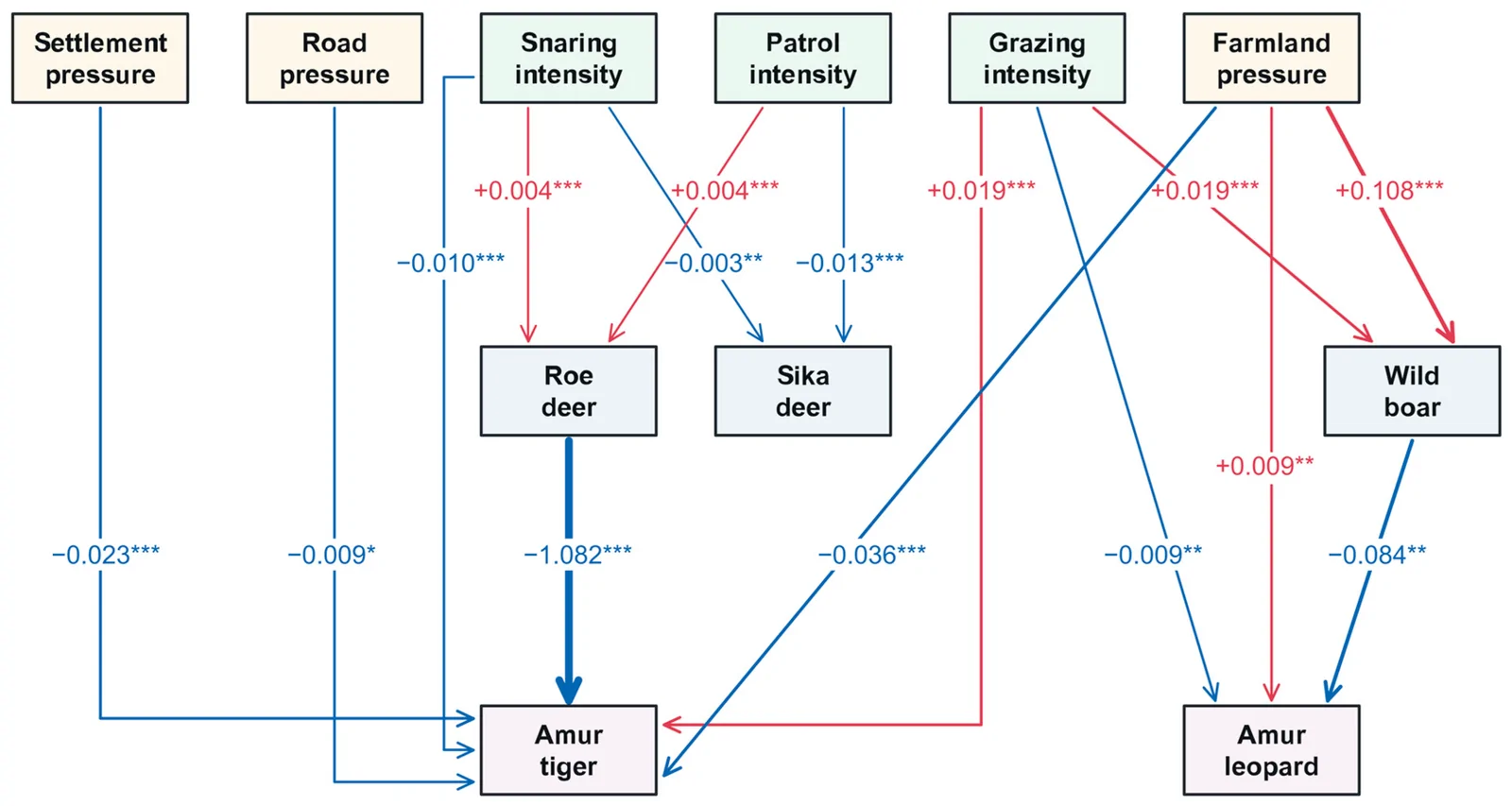
Submitted snow-free-season SEM diagram. Red and blue arrows indicate positive and negative associations, respectively. One, two and three asterisks indicate that the direction was consistent in at least 95.0%, 97.5% and 99.5% of posterior-state draws, respectively. These markers summarize the original state-only screen and are not conventional *p*-value significance markers or current interval support. Updated estimates are in Figure 8.

**Figure 8:**
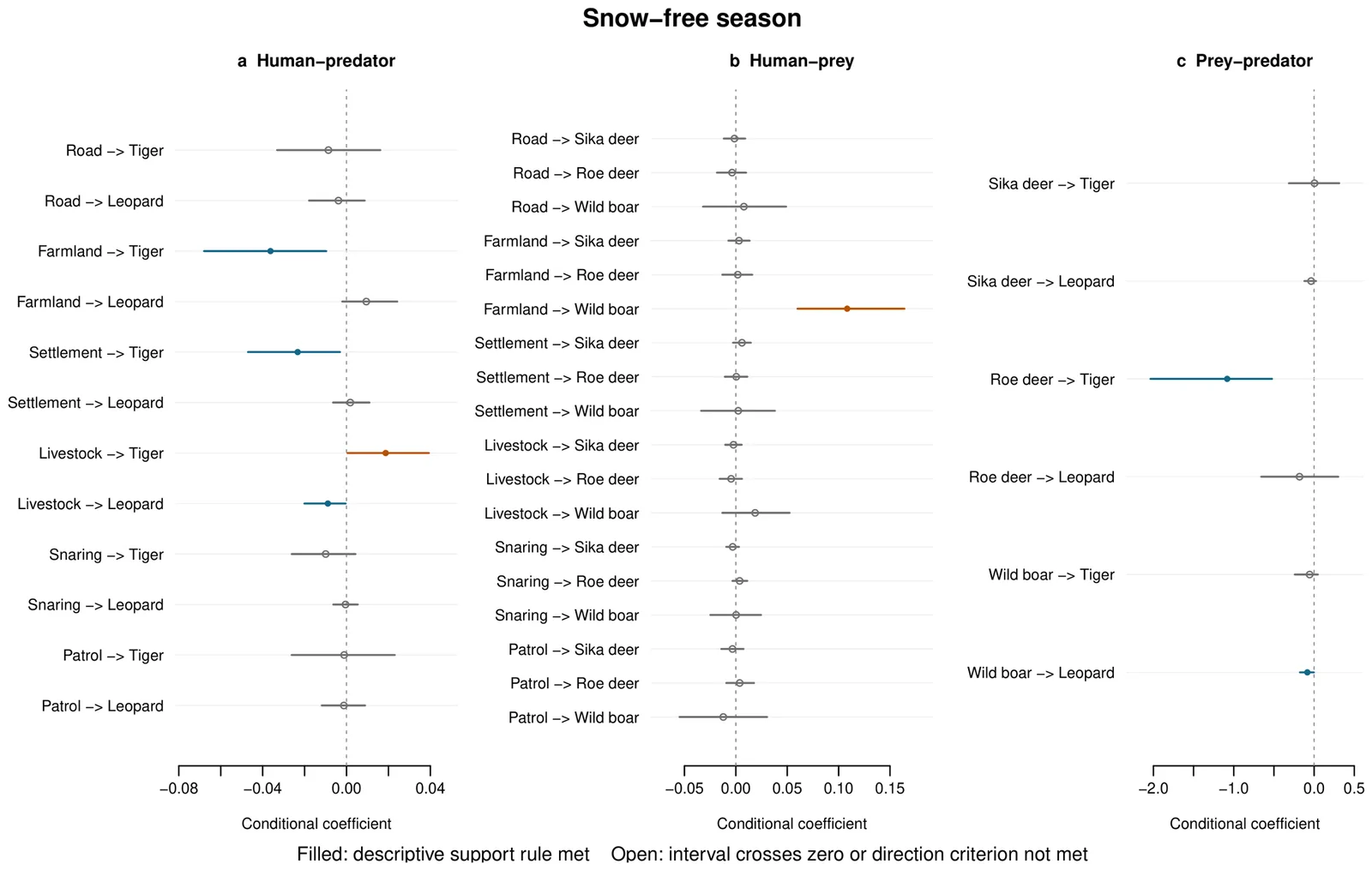
Park-wide conditional path estimates for the indicated season. Blue and orange points indicate negative and positive estimates, respectively. Points show medians of 500 state-conditioned estimates; bars show approximate 95% simulation intervals including state and within-equation coefficient uncertainty. Filled markers meet both descriptive screening criteria; open gray markers do not. Patrol intensity is a management covariate. Intervals are conditional on fitted spatial parameters and the specified network.

After including coefficient uncertainty, no direct human–predator path and two single-prey chains passed screening in snow; in snow-free conditions, four direct paths and one chain passed. The earlier state-only analysis screened 1/9 and 6/6 candidate paths, respectively. This change reflects sensitivity to uncertainty; counts describe screened pathways, not relative mediation strength. The original state-only seasonal diagrams and the updated park-wide conditional estimates are presented in numerical order in Figure 5, Figure 6, Figure 7 and Figure 8.

For H1, only the snow sika deer–tiger association supported the positive resource-tracking direction: 0.47747 (interval [0.19240,0.85227]). Roe deer–tiger coefficients were negative in both seasons, as was the snow-free boar–leopard coefficient. No winter leopard coefficient passed the extended rule.

For H2, none of the 15 pressure–prey comparisons per season supported the expected negative direction under the extended rule. Some farmland–prey and winter road–roe deer coefficients supported the opposite direction. H3 received support for snow-free farmland–tiger, settlement–tiger, and livestock–leopard associations, but not uniformly across pressures or predators. The livestock–tiger association was positive. The snow settlement–tiger interval now crosses zero: −0.01254 (interval [−0.02975,0.00187]). Tables S3 and S7 retain every comparison, including opposite and unresolved directions.

#### 3.3.2. Direct, Prey-Mediated, and Total Effects

H4 distinguishes a negative indirect product from the predicted sequence of component signs. The winter farmland–tiger net indirect estimate was −0.02212 (interval [−0.04478,−0.00517]), driven mainly by a positive farmland–roe deer association followed by a negative roe deer–tiger association. This sequence differs from the hypothesized negative pressure–prey and positive prey–predator links. The settlement–tiger and livestock–leopard net indirect intervals crossed zero. The snow-free snaring–tiger estimate also crossed zero (−0.00433; interval [−0.01367,0.00406]), so we do not report the state-only mediated percentage.

**Figure 9:**
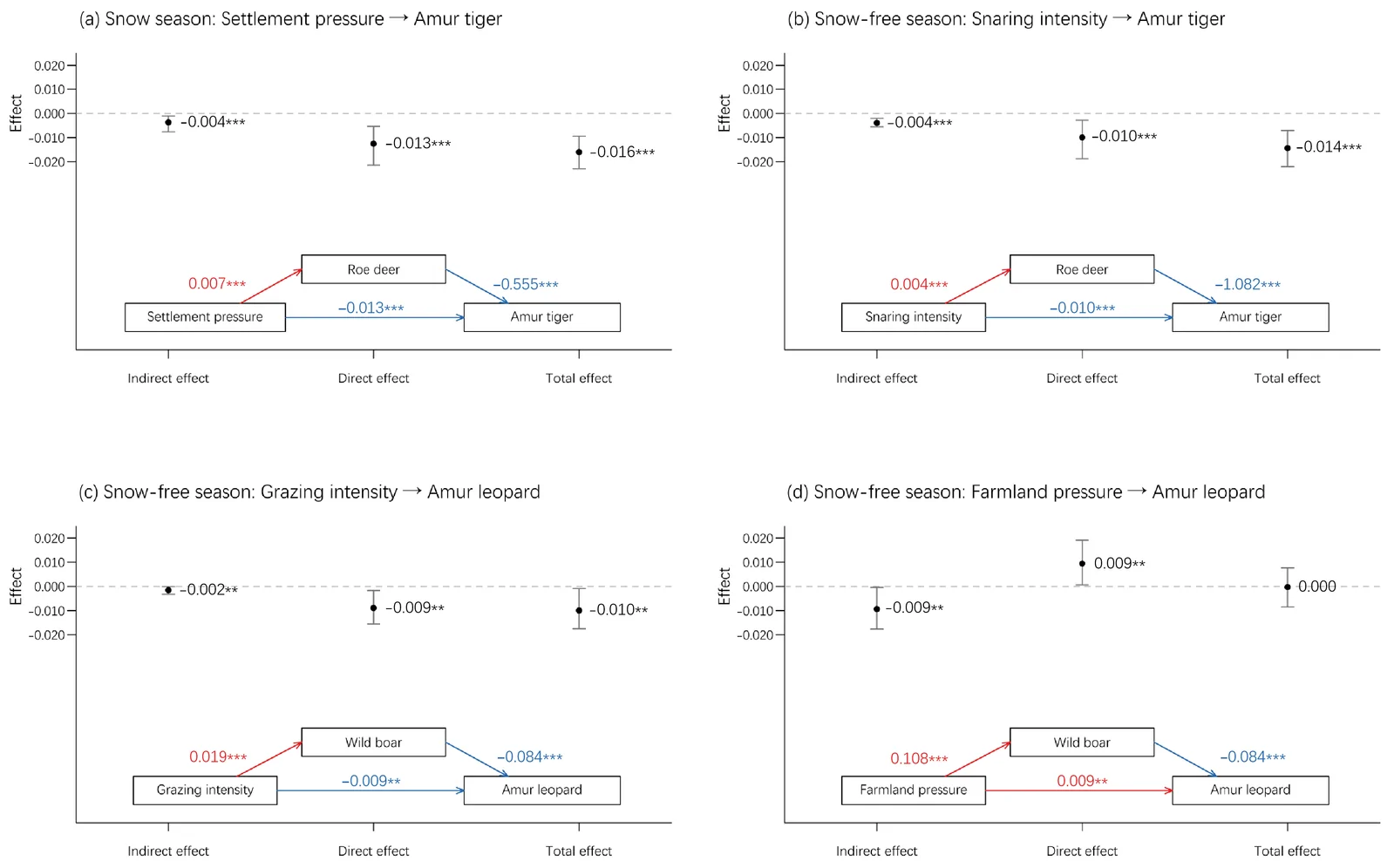
Original submitted decomposition for four illustrative pathways. Red and blue denote positive and negative associations, respectively. Two and three asterisks indicate at least 97.5% and 99.5% directional consistency across posterior-state draws; they are not conventional *p*-value significance markers. Labels, points, and intervals reflect the state-only analysis and exclude within-fit coefficient uncertainty. Updated selected-prey, net all-prey, and total associations are shown in Figure 10.

**Figure 10:**
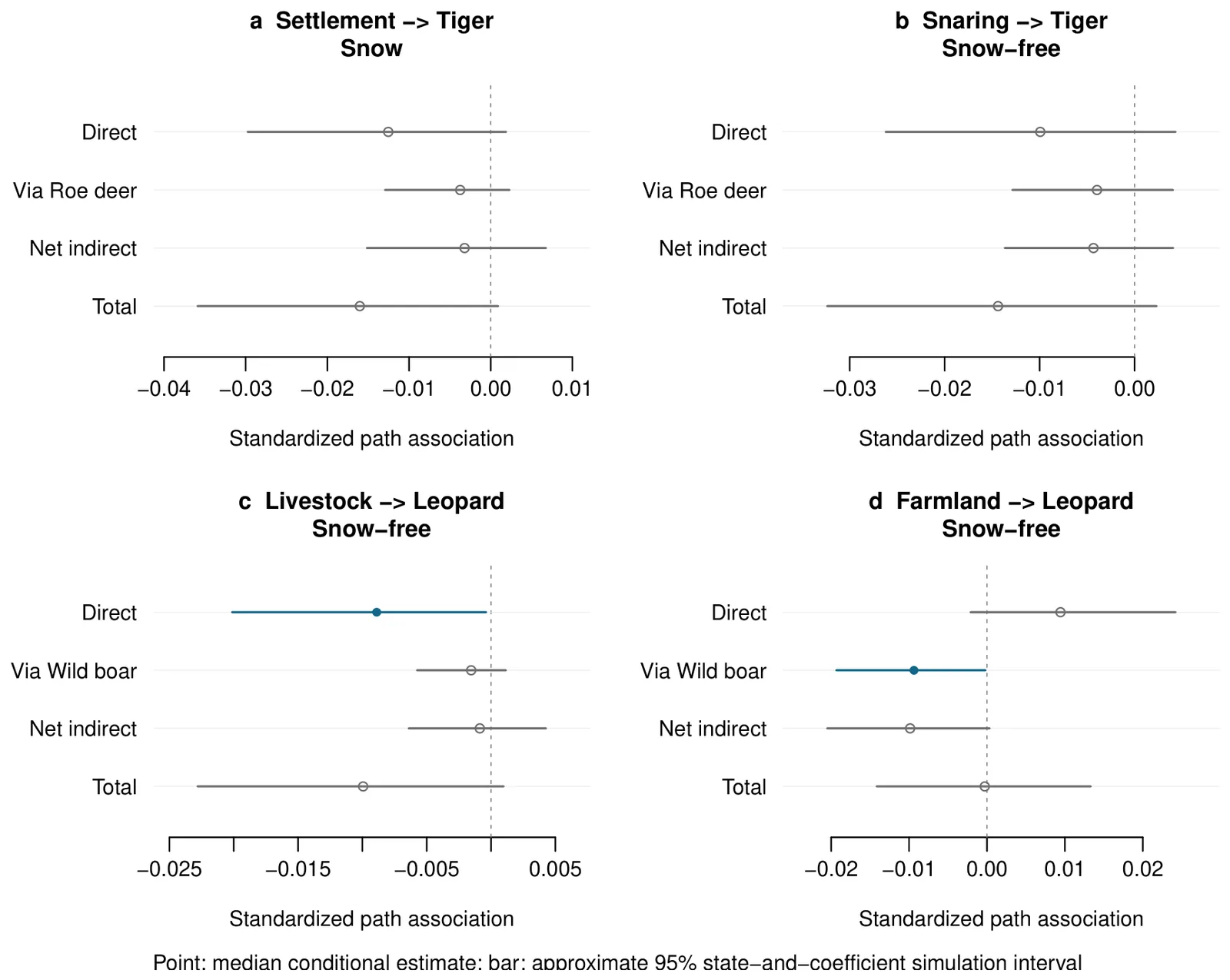
Direct, selected-prey indirect, net all-prey indirect, and total conditional associations for four illustrative cases. Blue points indicate negative effects that meet the extended descriptive support rule; this is a direction screen, not a conventional significance test. Points show medians; bars show approximate 95% state-and-coefficient simulation intervals. Axis ranges differ among panels. Selected-prey products differ from the net sum across prey. Intervals retain within-equation coefficient covariance and are conditional on the specified network.

The original state-only effect decomposition and the updated estimates incorporating coefficient uncertainty are shown in Figure 9 and Figure 10, respectively.

For snow-free farmland–leopard, direct and net indirect estimates had opposite signs, but the total was close to zero (−0.00028; interval [−0.01415,0.01330]). The interval leaves the total association unresolved; opposing point estimates alone do not establish cancellation. Table S3 reports all direct, prey-specific, net indirect, and total estimates.

#### 3.3.3. Spatial Structure and Model Sensitivity

Median Fisher’s *C* was 497.78 in snow and 158.06 in snow-free conditions. None of the winter fits and 3.0% of snow-free fits passed the 0.05 criterion (500 fits per season; eight degrees of freedom). Winter sika deer–roe deer independence was rejected in every draw; tiger–leopard independence was rejected in 91.6% of winter and 93.2% of snow-free fits. These fractions summarize repeated fit diagnostics, not posterior probabilities of ecological dependence (Tables S4 and S5).

Table S5 reports filtered-innovation Moran diagnostics and spatial-error parameters for all structural and independence-test equations. Residual spatial structure remained in some equations. Together with the Fisher’s *C* results, this indicates that the specified network leaves spatial and conditional relationships unexplained; individual paths should be interpreted within that network.

The alternative specifications did not improve overall network fit. Fisher’s *C* passed the 0.05 criterion in 0.0% of snow fits for the primary model and every 500-draw alternative; the single posterior-mean snow fit also failed. In snow-free conditions, passing fractions were 3.0% for the primary model, 0.6% for A1, 0.8% for A3, 0.0% for A4, and 22.4% for A5 and A6; the single A2 fit failed (Table S5e). These fractions summarize fit diagnostics across draws, not model probabilities.

A5 and A6 use the same three prey-pair independence claims, so their Fisher diagnostics are identical despite different predator specifications. Their higher passing fractions do not identify a tiger-to-leopard direction. A5 leaves the primary structural coefficients unchanged. Table S5f,g report tiger-to-leopard coefficients and residual-association intervals; these models estimate associations, not competition. Alternative spatial scales and weights remain to be evaluated.

### 3.4. B–H–M Spatial Classification and Pathway Heterogeneity

#### 3.4.1. B–H–M Classes and Spatial Patterns

In the B–H–M classification, high Biological value (*B*) occurred mainly in the south and southeast. High Human disturbance (*H*) was more common near park edges and areas of concentrated production and residential activity. Management context (*M*) broadly followed the current management zoning.

**Figure 11:**
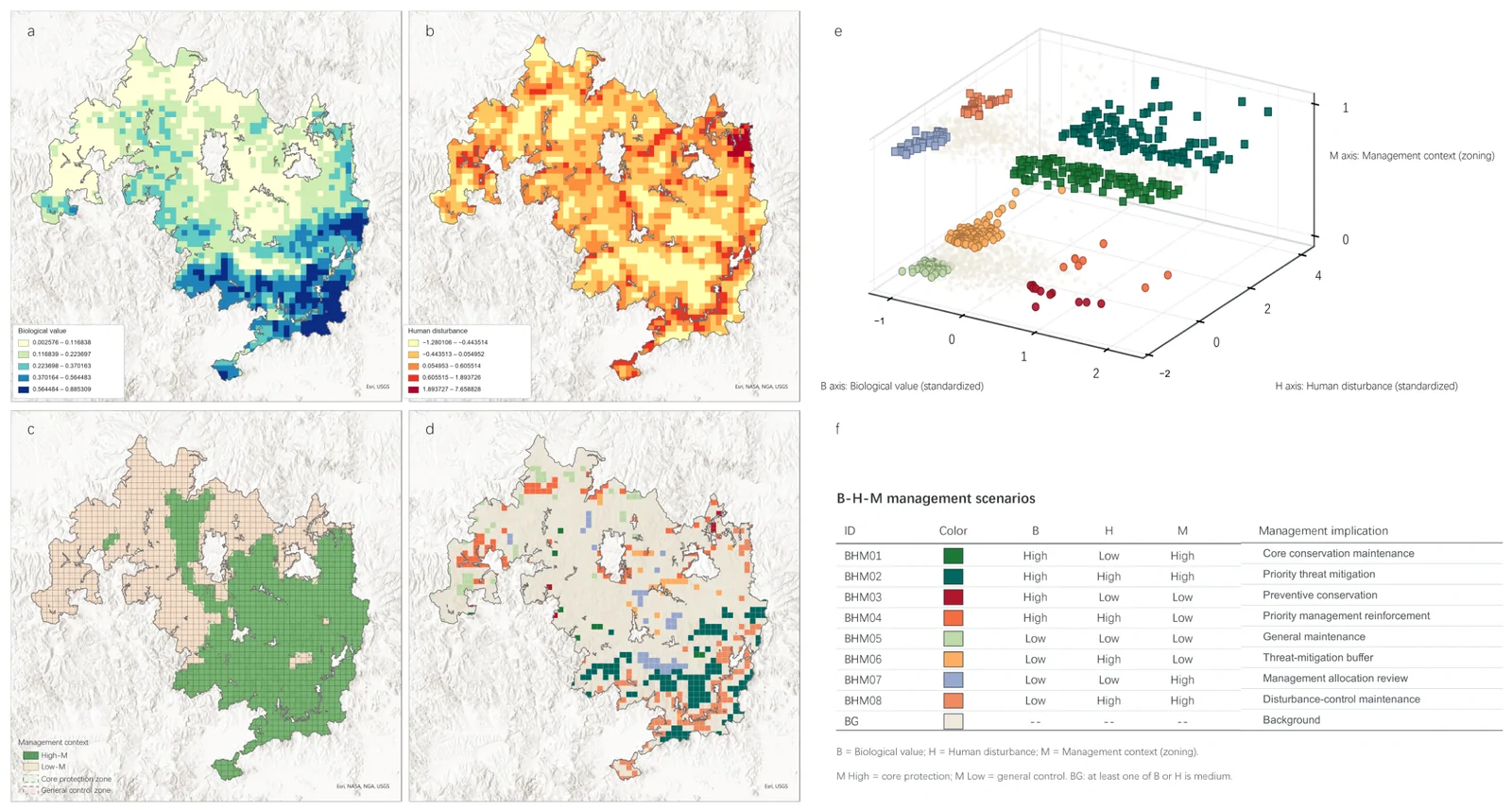
Spatial distribution. Panels (**a**–**f**) show: (**a**) biological value (B); (**b**) human disturbance (H); (**c**) management context (M); (**d**) the spatial distribution of B–H–M classes; (**e**) grid cells in standardized three-dimensional B–H–M space; and (**f**) scenario classes and their management implications. High M denotes the core protection zone and low M the general control zone; M is an institutional zoning context, not a measure of management success. Negative values use a true minus sign and numeric ranges use en dashes.

Combining the three axes produced eight management scenarios. High-*B* areas comprised preventive conservation, core conservation maintenance, priority management reinforcement, and priority threat mitigation zones, depending on *H* and *M*. Low-*B* areas comprised general maintenance, management allocation review, threat-mitigation buffer, and disturbance-control maintenance zones. The classification preserved distinct spatial combinations of conservation value, disturbance pressure, and management context. The B–H–M inputs, classified map, standardized three-dimensional distribution, and scenario key are summarized in Figure 11.

#### 3.4.2. Pathway Heterogeneity Across B–H–M Management Scenarios

The B–H–M diagrams show archived state-direction screening without within-fit coefficient uncertainty. These scenario results are exploratory and are not pooled with the extended park-wide intervals.

**Figure 12:**
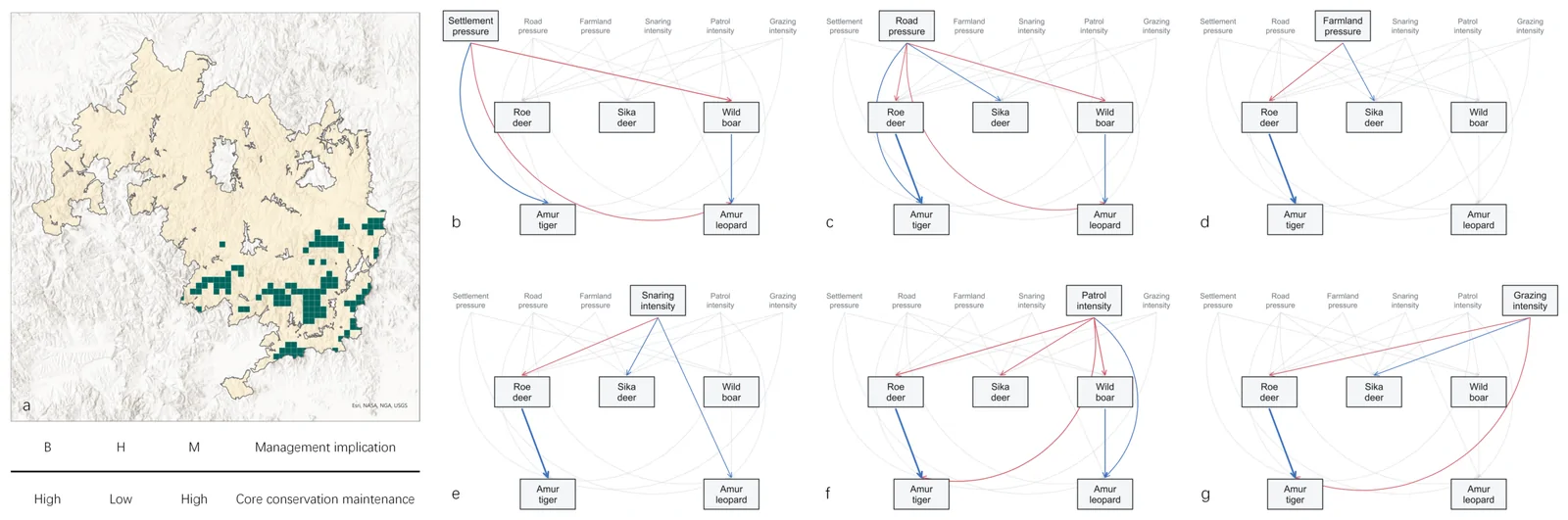
Original snow-free B–H–M diagram for the high-*B*–low-*H*–high-*M* core conservation maintenance context. Panel (**a**) maps the scenario; panels (**b**–**g**) highlight settlement, road, farmland, snaring, patrol, and livestock pathways in turn. Red and blue paths indicate positive and negative associations. Other variables and pathways remain gray for context; gray does not indicate a nonsignificant or unsupported pathway. Symbols show archived state-direction screening without within-fit coefficient uncertainty.

**Figure 13:**
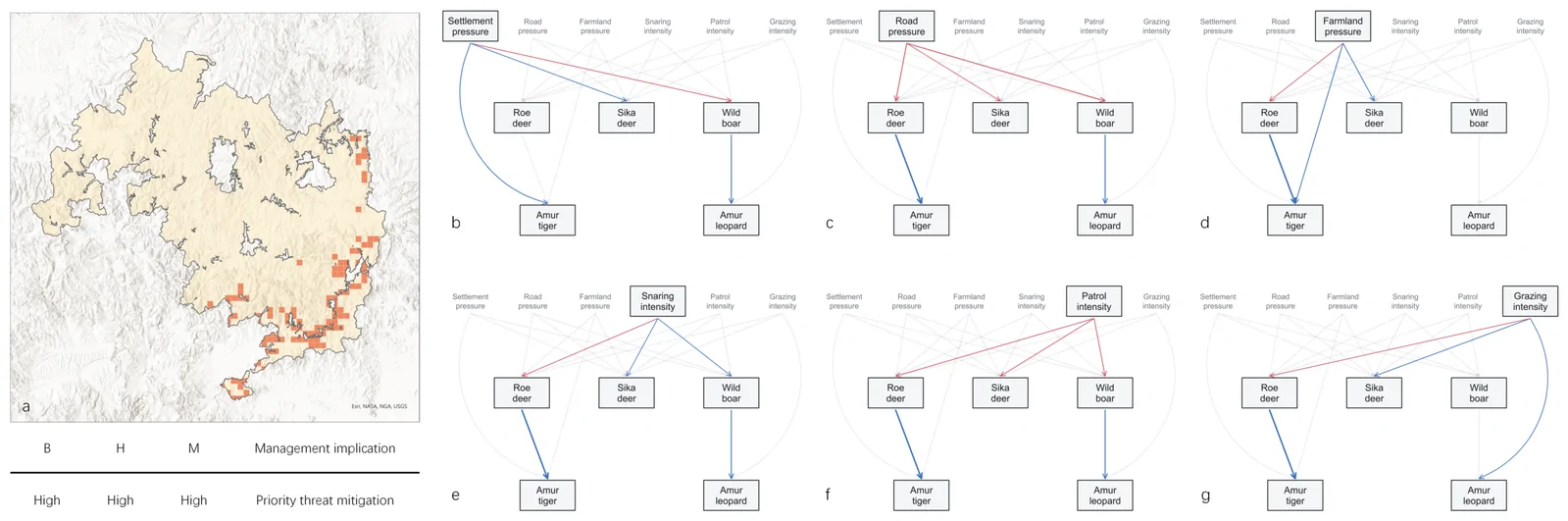
Original snow-free B–H–M diagram for the high-*B*–high-*H*–high-*M* priority threat mitigation context. Panel (**a**) maps the scenario; panels (**b**–**g**) highlight settlement, road, farmland, snaring, patrol, and livestock pathways in turn. Red and blue paths indicate positive and negative associations. Other variables and pathways remain gray for context; gray does not indicate a nonsignificant or unsupported pathway. Symbols show archived state-direction screening without within-fit coefficient uncertainty.

**Figure 14:**
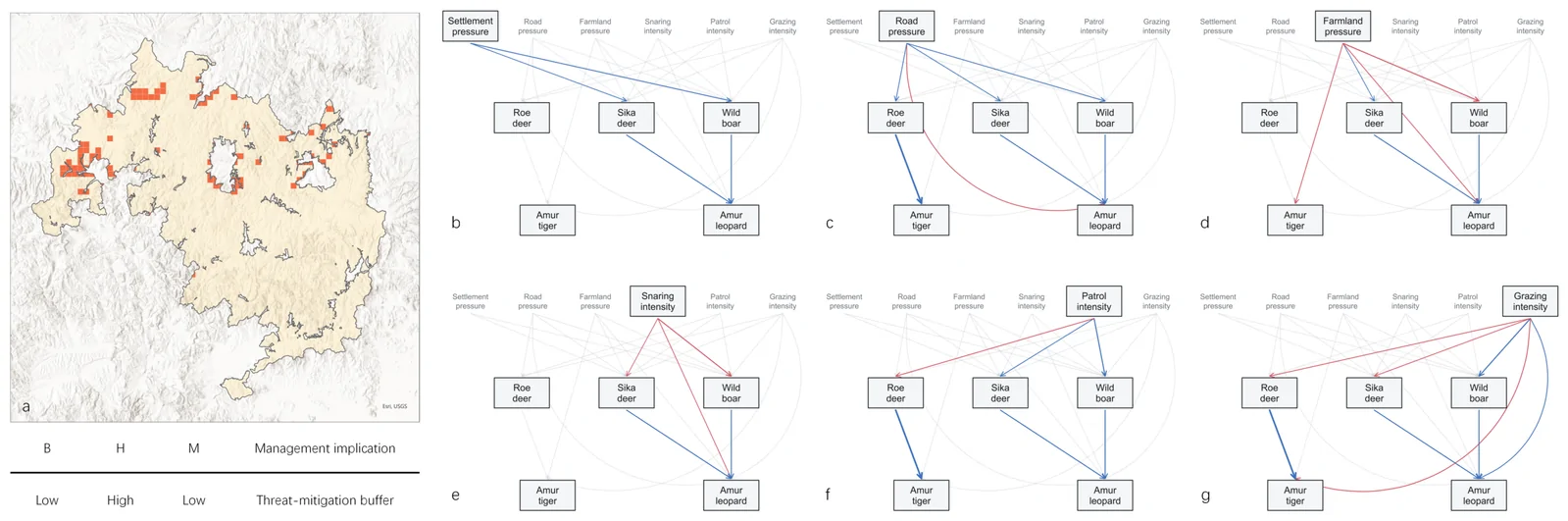
Original snow-free B–H–M diagram for the low-*B*–high-*H*–low-*M* threat-mitigation buffer context. Panel (**a**) maps the scenario; panels (**b**–**g**) highlight settlement, road, farmland, snaring, patrol, and livestock pathways in turn. Red and blue paths indicate positive and negative associations. Other variables and pathways remain gray for context; gray does not indicate a nonsignificant or unsupported pathway. Symbols show archived state-direction screening without within-fit coefficient uncertainty.

**Figure 15:**
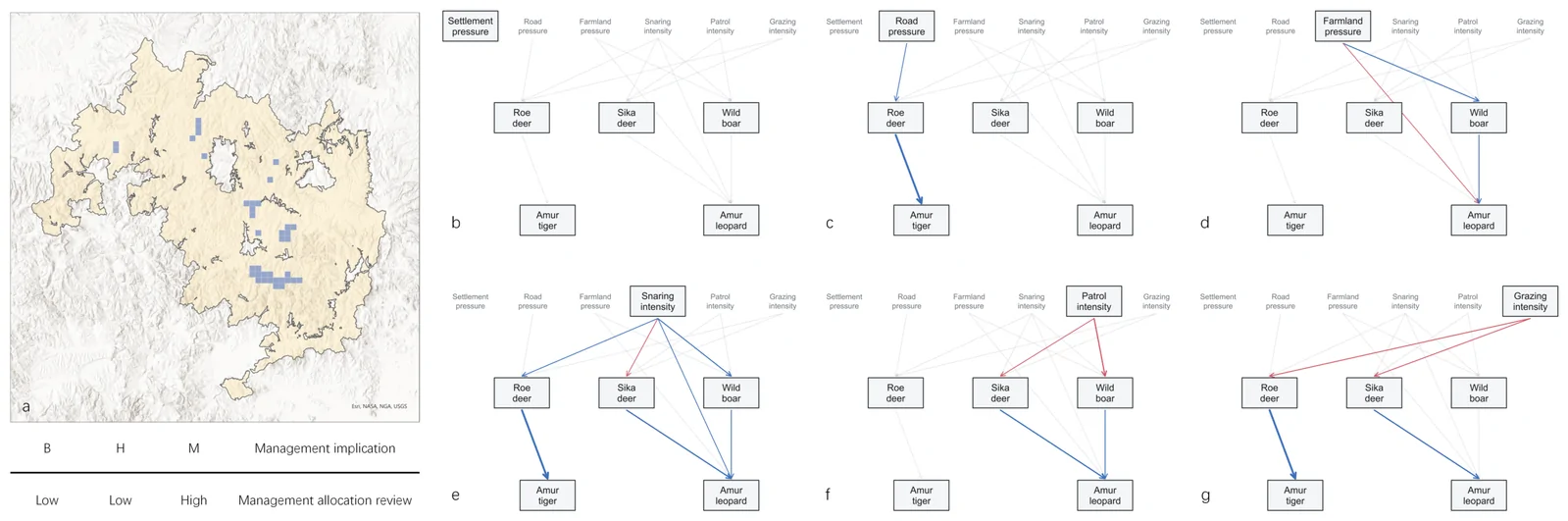
Original snow-free B–H–M diagram for the low-*B*–low-*H*–high-*M* management allocation review context. Panel (**a**) maps the scenario; panels (**b**–**g**) highlight settlement, road, farmland, snaring, patrol, and livestock pathways in turn. Red and blue paths indicate positive and negative associations. Other variables and pathways remain gray for context; gray does not indicate a nonsignificant or unsupported pathway. Symbols show archived state-direction screening without within-fit coefficient uncertainty.

We fitted snow-free-season spatial-error piecewise SEMs within each B–H–M scenario. Of the eight theoretical scenarios, four were reported as comparable because of limited sample sizes, insufficient within-scenario variation, and spatial structure. These were the high-*B*–high-*H*–high-*M* priority threat mitigation zone; the high-*B*–low-*H*–high-*M* core conservation maintenance zone; the low-*B*–high-*H*–low-*M* threat-mitigation buffer; and the low-*B*–low-*H*–high-*M* management allocation review zone (Figure 12, Figure 13, Figure 14 and Figure 15).

Some prey–predator pathways were consistent across scenarios. The negative conditional association between Siberian roe deer and Amur tigers was present in all four comparable scenarios. A negative wild boar–Amur leopard association also recurred. In contrast, the sika deer–Amur leopard pathway changed across B–H–M scenarios and had no consistent direction in low-*B* scenarios.

Human-disturbance pathways were more scenario-dependent. The road pressure–Siberian roe deer pathway was positive in both high-*B* scenarios but negative in both low-*B* scenarios. The number and direction of pathways involving farmland, snaring risk, patrols, and grazing also varied across scenarios. More pathways met the archived directional screen in the high-*B*–high-*H*–high-*M* scenario than in the low-*B*–low-*H*–high-*M* scenario.

Average park-wide SEM associations were not maintained across management scenarios. Some prey–predator directions recurred across archived state draws, whereas human disturbance–prey and human disturbance–predator pathways depended more strongly on the specific combination of conservation value, disturbance pressure, and management context.

## 4. Discussion

### 4.1. Seasonal Pathways and Predator Differences

Accounting for coefficient uncertainty reduced the number of pathways that passed screening, especially during snow. The seasonal contrast therefore concerns which conditional pathways were supported and how precisely they were estimated; it does not show stronger net mediation in winter or stronger direct disturbance effects in the snow-free season.

The snow sika deer–tiger association remained positive, while the snow settlement–tiger direct interval crossed zero. No leopard path passed screening in winter, despite more positive camera grid-months for leopard (310) than tiger (210). Leopard prey coefficients had broad intervals crossing zero (Methods S8). Restricted spatial variation, collinearity, and observation-model structure may also affect precision. Tigers were modeled with camera and patrol observations, whereas leopard models used cameras only, so species and data-source effects cannot be separated [8,9,57].

Previous studies suggest that predator responses to human disturbance are context-dependent. Hunchun tigers showed temporal avoidance of people without a direct spatial avoidance association, while some human associations operated through prey [13]. Other studies have reported seasonal shifts in species distribution and activity [11,12], associations between roads and tiger survival and foraging in the Russian Far East [14], and interactions among prey, disturbance, and tiger occurrence in Nepal and Thailand [58,59,60]. Livestock–deer–tiger regime shifts and human competition with African lions also show that disturbance relationships may vary among systems, without a shared threshold [61,62].

Predator interactions may also shape spatial associations. Food-web theory shows that predator interference can alter interaction strengths [63], and owl–canid vocal observations illustrate behavioral interactions in another assemblage [64]. The present grid-use data cannot test whether such interactions explain tiger–leopard associations.

### 4.2. Resource Predictions and Conditional Prey Associations

Across species, pressures, and seasons, H1–H4 were not supported as a common pathway. H1 was supported only for the snow sika deer–tiger association. H2 lacked support for a common negative pressure–prey relationship, while selected snow-free direct associations were consistent with H3. Some negative net indirect estimates arose from component signs opposite to H1 or H2, so they did not follow the proposed resource-depletion sequence in H4. Seasonal and B–H–M patterns differed descriptively, but H5 and H6 were not tested with formal coefficient contrasts.

Dietary importance and conditional spatial association address different questions. Diet studies identify prey consumed and selected; the multiprey SEM estimates each prey’s spatial gradient while accounting for other prey, disturbance, controls, and spatial error [9,10]. Negative coefficients may reflect habitat separation, prey composition, human exposure, or temporal mismatch. They describe spatial associations and do not imply that roe deer or wild boar populations should be reduced.

Human-modified edges may concentrate prey without necessarily providing equally suitable predator habitat. Crop-damage studies warrant further investigation of boar use near farmland and possible food subsidies [65,66]. In North China, disturbance types differed in their association with leopard–prey overlap [67]. Studies of temporal responses and human shielding further show that spatial co-occurrence alone cannot identify the mechanism [68,69].

### 4.3. Selected Prey Chains and Net Mediation

A negative product for one prey can be offset by pathways through other prey, so it need not produce a negative net association across all prey. Wider intervals in several examples show that direction-only screening understated uncertainty. We therefore report component signs, effect sizes, and approximate intervals without mediated percentages.

For farmland–leopard, the direct and net indirect estimates had opposite signs, but the total interval crossed zero. This pattern supports measuring both pathways before setting management priorities.

### 4.4. B–H–M Scenario Differences and Management Recommendations

The B–H–M map locates overlap between modeled predator use and measured human pressures within institutional zones. Because *M* represents zoning and *B* represents modeled use, these classes guide field assessment rather than estimate the effects of management changes.

Table 5 turns these spatial patterns into follow-up questions. The scenario diagrams retain the archived directional screen and remain exploratory; they are not pooled with the extended park-wide intervals.

The broader conservation literature discusses connectivity restoration, limiting land-use expansion, and patrol allocation as management options [14,43,45]. Their value in these scenarios should be evaluated against local field evidence and recorded outcomes.

Because *B* is derived from predator states, stratifying by *B* narrows the response range. Differences among scenario pathways may therefore reflect within-scenario variation and observation coverage as well as ecological heterogeneity. Evaluating zoning effectiveness requires actual management inputs and repeated outcomes.

### 4.5. Limits of Model Interpretation and Future Research

Poor Fisher’s *C* diagnostics indicate that the contemporaneous network does not fully capture conditional relationships, including prey-pair and predator-pair dependencies identified by the independence tests. Individual paths should therefore be read as conditional associations within the specified network.

Park-wide intervals include IDM state variation and approximate within-equation coefficient uncertainty; the livestock sensitivity propagates GAM mean-function uncertainty with spatial parameters fixed. Remaining uncertainties include uncertainty in the spatial parameters, residual imputation error, unknown missingness mechanisms, and B/H classification. Because operating logs were unavailable, camera-count proxies could not adjust for active operating days, camera-days, or failures, and the independent-detection interval could not be verified. Observed detections and modeled relative-use indices therefore should not be interpreted as abundance or density. Future monitoring should retain deployment, operation, and failure records to support duration-based effort adjustment. The six alternatives test selected model choices; alternative spatial weights, scales, and B–H–M thresholds remain to be evaluated.

Movement and kill-site observations, diet, and actual management inputs could distinguish resource availability from access, temporal avoidance, habitat separation, and unmeasured risk.

## 5. Conclusions

The spatial models did not support a common positive prey–predator or negative human-pressure pattern. Taken together, the conditional associations linking human disturbance, prey space use, and predator space use varied by species, pathway, and season, providing only partial support for the hypothesized patterns. Accounting for coefficient uncertainty reduced the number of supported park-wide pathways. Direct, prey-specific, net indirect, and total associations varied in sign and precision, and some negative products did not follow the hypothesized resource-depletion sequence.

The B–H–M classification identifies areas where modeled predator use overlaps with measured pressures, providing a spatial basis for setting management priorities and tailoring actions to local conditions. Follow-up should verify pressure sources in high-use areas, maintain comparable monitoring, and record management inputs alongside outcomes. These data will allow future studies to test whether targeted actions change human pressure, prey use, or predator use.

## Figures and Tables

**Table 1:** Data sources and quantification of human disturbance, environmental covariates, and animal observations used in this study.

Primary Data Type	Subcategory	Data Content	Quantification Metric	Original Format/Resolution	Year	Data Source/Product
**Human disturbance** **data**	Human land use and infrastructure	Settlements	Distance to settlements (m)	SHP	2022	BIGEMAP settlement data
		Farmland	Distance to farmland (m)	GeoTIFF	2023	Land-use/land-cover remote-sensing monitoring data
		Road network	Distance to roads (m)	SHP	2022	OpenStreetMap (Geofabrik China)
	Human activities	Livestock activity	Processed livestock detections per grid-month; standardized as livestock activity intensity	SHP/point records	2024–2025	Camera-trap data
		Patrol intensity	Patrol route length per grid-month (km), standardized as patrol intensity	SHP/patrol tracks	2024–2025	SMART patrol data
		Snaring records	Snare/trap records per grid-month, standardized as snaring intensity	SHP/point records	2024–2025	SMART patrol data
**Natural** **environmental data**	Terrain	Digital elevation model (DEM)	Elevation (m)	GeoTIFF/30 m	2000	NASA DEM via OpenTopography
		Slope	Slope (°)	GeoTIFF/30 m	2000	Derived from DEM
		Terrain ruggedness	Terrain Ruggedness Index (TRI)	GeoTIFF/30 m	2000	Derived from DEM
	Forest and vegetation	NDVI	Mean and maximum NDVI (NDVI_Mean, NDVI_Max)	Remote-sensing raster	2024	NASA AppEEARS
		Canopy height	Mean canopy height (m)	GeoTIFF/10 m	2020	Global Canopy Height 2020
		Canopy closure	Mean canopy closure (%)	SHP/forest stand	2024	Local natural background survey
		Mean DBH	Mean diameter at breast height (cm)	SHP/forest stand	2024	Local natural background survey
		Basal area	Stand basal area (m^2^ ha^−1^)	SHP/forest stand	2024	Derived from local natural background survey
		Forest edge	Distance to forest edge (m)	SHP/forest stand	2024	Derived from forest-stand boundaries
		Primary forest	Proportion of primary-forest cover	SHP/forest stand	2024	Local natural background survey
	Hydrology	Permanent water bodies	Distance to permanent water (m)	SHP	2022	OpenStreetMap (Geofabrik China)
	Snow and seasonal environment	Snow depth	Monthly snow-depth/snow-water conditions	GeoTIFF/∼9 km	2024	ERA5-Land monthly averaged reanalysis
		Snow-cover frequency	Snow-cover persistence/frequency	GeoTIFF/500 m	2024	MODIS/Terra CGF Snow Cover Daily L3, Version 61
		Composite snow pressure	Composite snow-pressure index (snow_pressure_main_01)	Resampled and aggregated raster	2024	Derived from ERA5-Land and MODIS snow variables
**Animal observation** **data**	Camera-trap data	Amur tiger, Amur leopard, sika deer, Siberian roe deer, and wild boar	Monthly detection/non-detection for predators; processed detection counts for prey	SHP/camera locations	Predators: 2024–2025; prey: 2024	Northeast China Tiger and Leopard National Park camera-trap monitoring data
	SMART patrol data	Tiger and three prey species included in fitted IDM; leopard patrol data excluded	Monthly detection/non-detection; patrol distance used to account for observation effort	SHP/points and tracks	Predators: 2024–2025; prey: 2024	Northeast China Tiger and Leopard National Park SMART patrol data

*Note*: Source records cover 2024–2025 for predators and 2024 for prey; archived formal model inputs cover January–December 2024. All spatial data were projected to WGS 84/UTM Zone 52N and first summarized using a 500 m base grid. After spatial-scale evaluation, variables were aggregated to the 3 km grid used in the main analyses. DEM, digital elevation model; TRI, Terrain Ruggedness Index; NDVI, normalized difference vegetation index; DBH, diameter at breast height; SMART, Spatial Monitoring and Reporting Tool.

**Table 3:** Comparison of model suitability across spatial scales.

Metric	1km	3km	5km
$N_{\mathrm{site}}$	14,742	1824	711
Detection-positive rate	3.60%	12.49%	20.65%
$\hat{R}$	1.0026–1.2998	1.0009–1.0229	1.0026–1.0295
$n_{\mathrm{eff}}$ or ESS	11–1500	118–1253	77–630
$\bar{\psi }$	–	0.217	0.219
AUC	0.775	0.832	0.806

**Table 4:** Final environmental covariate assignments in the archived predator V6 and prey V3 state models.

Species	Season	State Component	Covariates
Amur tiger	Both	Spatial-use probability	water distance, canopy height, elevation, mean DBH, slope
Amur leopard	Both	Spatial-use probability	elevation, basal area, water distance, primary forest, mean DBH
Sika deer	Snow	Latent use and intensity	water distance, edge distance, terrain ruggedness, elevation, mean DBH, snow pressure
Siberian roe deer	Snow	Intensity; latent use intercept only	primary forest, canopy height, basal area, mean DBH, elevation, snow pressure
Wild boar	Snow	Latent use and intensity	canopy closure, elevation, terrain ruggedness, water distance, mean DBH, snow pressure
Sika deer	Snow-free	Latent use and intensity	water distance, edge distance, terrain ruggedness, elevation, mean DBH
Siberian roe deer	Snow-free	Intensity; latent use intercept only	primary forest, canopy height, basal area, mean DBH, elevation
Wild boar	Snow-free	Latent use and intensity	canopy closure, elevation, terrain ruggedness, water distance, mean DBH

Both predator models include the snow-season intercept contrast and snow-pressure term. The leopard model additionally includes an elevation-by-snow interaction; the tiger model has no such covariate interaction. Prey seasons are fitted separately. DBH, diameter at breast height. Species/season masks and archived variable codes are retained in Table S2a,b.

**Table 5:** Observed patterns and follow-up checks for the four reported B–H–M contexts.

Context	Observation Supported by the Classification	Follow-Up Assessment
High *B*–high *H*–high *M* Priority threat mitigation	High modeled predator use and measured pressure overlap within core protection zoning.	Verify the locations and sources of measured pressures; collect actual management-input and repeated outcome records.
High *B*–low *H*–high *M* Core conservation maintenance	High modeled use co-occurs with relatively low measured pressure.	Check whether low pressure persists through repeated, comparable monitoring and assess unmeasured exposure.
Low *B*–high *H*–low *M* Threat-mitigation buffer	Low modeled use and high measured pressure co-occur in general control zoning.	Investigate detection coverage and habitat use before attributing low modeled use to disturbance or assigning recovery functions.
Low *B*–low *H*–high *M* Management allocation review	Low modeled use and measured pressure co-occur within core protection zoning.	Verify sampling coverage, seasonal use and actual inputs before drawing conclusions about resource allocation.

## Data Availability

The data presented in this study are available upon reasonable request from the corresponding author. The data are not publicly available because they contain sensitive locations of threatened species, camera-trap sites, and ranger patrol information; access is subject to approval by the relevant data-owning and park management authorities.

## Supplementary Materials

The following supporting information can be downloaded at: https://www.mdpi.com/article/10.3390/ani16213480/s1, Supplementary Methods and Numerical Results, including the initial network, Tables S1–S8, and numerical source tables for the primary model and six park-wide alternatives. Reference [70] is cited in the supplementary materials.
