# Supplementary material for: Mechanisms Linking Human Activities to Habitat Use by Amur Tigers and Amur Leopards Under Differentiated Management
Source: Animals (Basel). 2026 Nov 3;16(21):3480. doi: 10.3390/ani16213480 (PMC13761537; doi:10.3390/ani16213480)
Supplement: Supplementary file 1 [file animals-16-03480-s001.zip › animals-4620352-supplementary.pdf]

Supplementary Methods and Numerical Results

The tables distinguish the retained archived fits from revision-stage uncertainty assessment. Park-wide primary coefficient and decomposition intervals in Table S3 combine latent-state variation with approximate conditional coefficient uncertainty. Tables S4–S5 document archived within-fit diagnostics and alternative-specification summaries; their state-only intervals retain that label. B–H–M numerical results are unchanged. Current numerical source tables have the prefix `v5_`; earlier tables document the archived analyses rather than the new uncertainty intervals.

Figure S1 Initial hypothesized path network

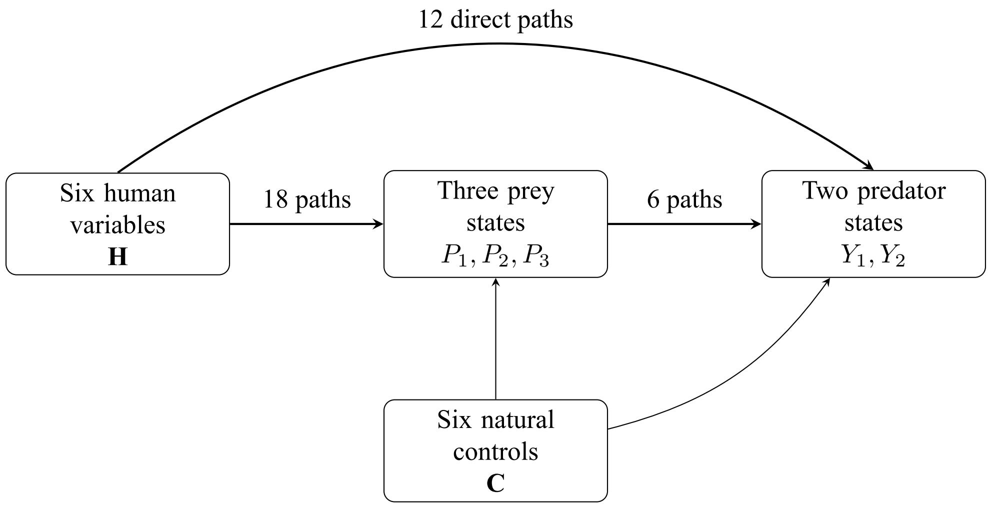

Grouped arrows indicate that all variables in the source group enter every local target equation. Natural controls enter each prey and predator equation. The main model has no predator-to-predator path or correlated innovation. Spatial error dependence is modeled within each equation. Arrow direction specifies the fitted conditional model and is not an identified causal direction.

Table S1a Predator source-specific grid-month observations

Species	Season	Source	Valid	Positive	Zero	Missing
Tiger	Snow	camera	4560	210	4350	10032
Tiger	Snow	patrol	2438	10	2428	12154
Tiger	Snow-free	camera	9120	279	8841	5472
Tiger	Snow-free	patrol	2114	7	2107	12478
Leopard	Snow	camera	4560	310	4250	10032
Leopard	Snow	patrol	0	0	0	14592
Leopard	Snow-free	camera	9120	448	8672	5472
Leopard	Snow-free	patrol	0	0	0	14592

Missing counts include padded predator visits as well as unavailable source observations. Leopard patrol data were excluded. Positive grid-months are not counts of individual animals.

Table S1b Prey observation coverage and processed counts

Species	Season	Source	Valid	Positive	Record sum	Missing
Deer	Snow	Camera count	4560	2242	23199	2736

Species	Season	Source	Valid	Positive	Record sum	Missing
Deer	Snow	Patrol binary	2457	101	101	4839
Roe	Snow	Camera count	4560	3685	55170	2736
Roe	Snow	Patrol binary	2650	618	618	4646
Boar	Snow	Camera count	4560	2170	9903	2736
Boar	Snow	Patrol binary	2524	306	306	4772
Deer	Snow-free	Camera count	9120	5871	130161	5472
Deer	Snow-free	Patrol binary	2243	216	216	12349
Roe	Snow-free	Camera count	9120	7507	155485	5472
Roe	Snow-free	Patrol binary	2620	925	925	11972
Boar	Snow-free	Camera count	9120	4894	24548	5472
Boar	Snow-free	Patrol binary	2232	299	299	12360

Camera record sums and patrol-positive visit sums differ in observational unit.

Table S1c Composition of valid source-specific observations

Species	Camera units	Patrol units	Camera percent	Patrol percent
Tiger	13680	4552	75.03	24.97
Leopard	13680	0	100.00	0.00
Deer	13680	4700	74.43	25.57
Roe	13680	5270	72.19	27.81
Boar	13680	4756	74.20	25.80

Denominator is camera plus patrol valid species–grid–month observations, summed across the two seasons. These percentages describe input composition, not model weights or independent information.

Table S2 Prey state-process covariate assignments

Season	Species	Component	Slot	Variable
Snow	Deer	psi_occupancy	1	dist_water
Snow	Deer	psi_occupancy	2	dist_edge
Snow	Deer	psi_occupancy	3	TRI
Snow	Deer	psi_occupancy	4	DEM
Snow	Deer	psi_occupancy	5	xiong_jing
Snow	Deer	psi_occupancy	6	snow_pressure_main_01
Snow	Deer	lambda_intensity	1	dist_water
Snow	Deer	lambda_intensity	2	dist_edge
Snow	Deer	lambda_intensity	3	TRI
Snow	Deer	lambda_intensity	4	DEM
Snow	Deer	lambda_intensity	5	xiong_jing
Snow	Deer	lambda_intensity	6	snow_pressure_main_01
Snow	Roe	lambda_intensity	1	orig_cov_prop
Snow	Roe	lambda_intensity	2	guan_ceng_gao_du
Snow	Roe	lambda_intensity	3	ji_mian
Snow	Roe	lambda_intensity	4	xiong_jing
Snow	Roe	lambda_intensity	5	DEM
Snow	Roe	lambda_intensity	6	snow_pressure_main_01
Snow	Boar	psi_occupancy	1	yu_bi_du

Season	Species	Component	Slot	Variable
Snow	Boar	psi_occupancy	2	DEM
Snow	Boar	psi_occupancy	3	TRI
Snow	Boar	psi_occupancy	4	dist_water
Snow	Boar	psi_occupancy	5	xiong_jing
Snow	Boar	psi_occupancy	6	snow_pressure_main_01
Snow	Boar	lambda_intensity	1	yu_bi_du
Snow	Boar	lambda_intensity	2	DEM
Snow	Boar	lambda_intensity	3	TRI
Snow	Boar	lambda_intensity	4	dist_water
Snow	Boar	lambda_intensity	5	xiong_jing
Snow	Boar	lambda_intensity	6	snow_pressure_main_01
Snow-free	Deer	psi_occupancy	1	dist_water
Snow-free	Deer	psi_occupancy	2	dist_edge
Snow-free	Deer	psi_occupancy	3	TRI
Snow-free	Deer	psi_occupancy	4	DEM
Snow-free	Deer	psi_occupancy	5	xiong_jing
Snow-free	Deer	lambda_intensity	1	dist_water
Snow-free	Deer	lambda_intensity	2	dist_edge
Snow-free	Deer	lambda_intensity	3	TRI
Snow-free	Deer	lambda_intensity	4	DEM
Snow-free	Deer	lambda_intensity	5	xiong_jing
Snow-free	Roe	lambda_intensity	1	orig_cov_prop
Snow-free	Roe	lambda_intensity	2	guan_ceng_gao_du
Snow-free	Roe	lambda_intensity	3	ji_mian
Snow-free	Roe	lambda_intensity	4	xiong_jing
Snow-free	Roe	lambda_intensity	5	DEM
Snow-free	Boar	psi_occupancy	1	yu_bi_du
Snow-free	Boar	psi_occupancy	2	DEM
Snow-free	Boar	psi_occupancy	3	TRI
Snow-free	Boar	psi_occupancy	4	dist_water
Snow-free	Boar	psi_occupancy	5	xiong_jing
Snow-free	Boar	lambda_intensity	1	yu_bi_du
Snow-free	Boar	lambda_intensity	2	DEM
Snow-free	Boar	lambda_intensity	3	TRI
Snow-free	Boar	lambda_intensity	4	dist_water
Snow-free	Boar	lambda_intensity	5	xiong_jing

Table S2b Predator state-process covariate assignments

Species	Component	Archived variable
Tiger	common_state	dist_water
Tiger	common_state	guan_ceng_gao_du
Tiger	common_state	DEM
Tiger	common_state	xiong_jing
Tiger	common_state	Slope
Tiger	snow_pressure	snow_pressure_main_01
Leopard	common_state	DEM
Leopard	common_state	ji_mian
Leopard	common_state	dist_water
Leopard	common_state	orig_cov_prop
Leopard	common_state	xiong_jing
Leopard	snow_interaction	DEM

Species	Component	Archived variable
Leopard	snow_pressure	snow_pressure_main_01

Common-state coefficients apply to both seasons, with a separate snow-season intercept contrast. Snow pressure is included during snow for both predators; only leopard elevation has the additional snow interaction. Archived names correspond to water/edge distance, elevation, slope, terrain ruggedness, mean DBH (xiong_jing), basal area (ji_mian), canopy closure (yu_bi_du), canopy height (guan_ceng_gao_du), primary forest (orig_cov_prop), and composite snow pressure, as applicable to the species-specific masks.

Table S3a Snow full component coefficients

Points are medians across the 500 original conditional estimates. Lower and upper bounds are the 2.5th and 97.5th percentiles of 200,000 state-and-coefficient simulations. Direction is the dominant sign fraction, not a posterior model probability. Y denotes the descriptive dual support rule. Natural controls are included for completeness.

Response	Predictor	Median	Lower	Upper	Direction	Rule
Sika deer	Road pressure	0.01427	-0.00165	0.02985	0.9608	–
Sika deer	Farmland pressure	-0.00303	-0.01898	0.01180	0.6484	–
Sika deer	Settlement pressure	0.00290	-0.01011	0.01556	0.6860	–
Sika deer	Livestock activity	0.00119	-0.00774	0.01054	0.5990	–
Sika deer	Snaring	-0.00565	-0.01728	0.00485	0.8481	–
Sika deer	Patrol intensity	-0.00792	-0.02038	0.00462	0.8957	–
Sika deer	Elevation	-0.88575	-0.97656	-0.79269	1.0000	Y
Sika deer	Mean DBH	0.26277	0.15957	0.38136	1.0000	Y
Sika deer	Edge distance	-0.04398	-0.11155	0.03191	0.8630	–
Sika deer	Water distance	0.03067	-0.07544	0.14197	0.7206	–
Sika deer	NDVI	0.01571	-0.00173	0.03251	0.9620	–
Sika deer	Snow pressure	0.09113	-0.02452	0.23327	0.9363	–
Roe deer	Road pressure	0.01489	0.00076	0.03003	0.9807	Y
Roe deer	Farmland pressure	0.02126	0.00637	0.03822	0.9983	Y
Roe deer	Settlement pressure	0.00661	-0.00431	0.01820	0.8840	–
Roe deer	Livestock activity	0.00250	-0.00553	0.01089	0.7277	–
Roe deer	Snaring	0.00575	-0.00417	0.01667	0.8680	–
Roe deer	Patrol intensity	-0.00031	-0.01120	0.01079	0.5260	–
Roe deer	Elevation	0.82987	0.69717	0.95743	1.0000	Y
Roe deer	Mean DBH	-0.13594	-0.24300	-0.03951	0.9978	Y
Roe deer	Edge distance	0.00515	-0.00580	0.01706	0.8154	–
Roe deer	Water distance	0.00086	-0.02611	0.02995	0.5234	–
Roe deer	NDVI	-0.01871	-0.05715	0.01921	0.8248	–
Roe deer	Snow pressure	0.37816	0.20516	0.52493	1.0000	Y
Wild boar	Road pressure	0.00438	-0.02002	0.02555	0.6941	–
Wild boar	Farmland pressure	0.06650	0.00899	0.12267	0.9913	Y
Wild boar	Settlement pressure	0.00239	-0.02036	0.02332	0.6080	–
Wild boar	Livestock activity	-0.00022	-0.01442	0.01491	0.5270	–
Wild boar	Snaring	0.01249	-0.00281	0.03198	0.9469	–
Wild boar	Patrol intensity	-0.01690	-0.03945	0.00026	0.9735	–
Wild boar	Elevation	-0.40203	-0.75180	-0.09132	0.9948	Y
Wild boar	Mean DBH	-0.47851	-0.70719	-0.20267	1.0000	Y
Wild boar	Edge distance	0.00864	-0.01078	0.03129	0.8211	–
Wild boar	Water distance	-0.22339	-0.47450	0.03741	0.9586	–
Wild boar	NDVI	-0.07998	-0.16917	0.01079	0.9540	–
Wild boar	Snow pressure	-0.16599	-0.46781	0.21735	0.8085	–

Response	Predictor	Median	Lower	Upper	Direction	Rule
Tiger	Sika deer	0.47747	0.19240	0.85227	0.9999	Y
Tiger	Roe deer	-0.55458	-1.29236	-0.16861	0.9979	Y
Tiger	Wild boar	-0.13161	-0.67490	0.02557	0.9450	–
Tiger	Road pressure	-0.00122	-0.01964	0.01692	0.5379	–
Tiger	Farmland pressure	-0.00858	-0.03282	0.01041	0.8095	–
Tiger	Settlement pressure	-0.01254	-0.02975	0.00187	0.9553	–
Tiger	Livestock activity	0.00183	-0.00843	0.01233	0.6465	–
Tiger	Snaring	0.00232	-0.01014	0.01508	0.6531	–
Tiger	Patrol intensity	-0.00288	-0.01811	0.01195	0.6525	–
Tiger	Elevation	0.45525	-0.14727	1.28959	0.9270	–
Tiger	Mean DBH	0.11450	-0.20649	0.36151	0.8088	–
Tiger	Edge distance	0.02385	-0.01243	0.07237	0.8999	–
Tiger	Water distance	-0.04772	-0.28097	0.11783	0.7125	–
Tiger	NDVI	0.11740	0.05690	0.18655	1.0000	Y
Tiger	Snow pressure	-0.26360	-0.56331	0.18271	0.8744	–
Leopard	Sika deer	-0.10265	-0.24480	0.00610	0.9672	–
Leopard	Roe deer	0.26832	-0.09535	0.69628	0.9266	–
Leopard	Wild boar	-0.06488	-0.23063	0.03760	0.9096	–
Leopard	Road pressure	-0.00523	-0.01621	0.00365	0.8468	–
Leopard	Farmland pressure	-0.00156	-0.01093	0.01181	0.6146	–
Leopard	Settlement pressure	0.00189	-0.00496	0.00968	0.7131	–
Leopard	Livestock activity	-0.00098	-0.00631	0.00372	0.6512	–
Leopard	Snaring	-0.00410	-0.01177	0.00231	0.8790	–
Leopard	Patrol intensity	-0.00293	-0.01094	0.00357	0.7862	–
Leopard	Elevation	-0.62220	-1.15318	-0.16860	0.9957	Y
Leopard	Mean DBH	0.63523	0.47321	0.85844	1.0000	Y
Leopard	Edge distance	-0.00325	-0.01954	0.00652	0.7500	–
Leopard	Water distance	-0.04252	-0.15154	0.06645	0.7945	–
Leopard	NDVI	0.01700	-0.00232	0.04428	0.9566	–
Leopard	Snow pressure	-0.68026	-0.89253	-0.42371	1.0000	Y

Table S3b Snow full path decomposition

Predator	Human variable	Quantity	Median	Lower	Upper
Tiger	Road pressure	direct	-0.00122	-0.01964	0.01692
Tiger	Road pressure	net indirect	-0.00246	-0.01798	0.01103
Tiger	Road pressure	total	-0.00396	-0.02462	0.01608
Tiger	Farmland pressure	direct	-0.00858	-0.03282	0.01041
Tiger	Farmland pressure	net indirect	-0.02212	-0.04478	-0.00517
Tiger	Farmland pressure	total	-0.03185	-0.05813	-0.01050
Tiger	Settlement pressure	direct	-0.01254	-0.02975	0.00187
Tiger	Settlement pressure	net indirect	-0.00320	-0.01516	0.00675
Tiger	Settlement pressure	total	-0.01603	-0.03589	0.00090
Tiger	Livestock activity	direct	0.00183	-0.00843	0.01233
Tiger	Livestock activity	net indirect	-0.00058	-0.00825	0.00680
Tiger	Livestock activity	total	0.00135	-0.01130	0.01408
Tiger	Snaring	direct	0.00232	-0.01014	0.01508
Tiger	Snaring	net indirect	-0.00787	-0.01883	0.00053
Tiger	Snaring	total	-0.00555	-0.02087	0.00882
Tiger	Patrol intensity	direct	-0.00288	-0.01811	0.01195

Predator	Human variable	Quantity	Median	Lower	Upper
Tiger	Patrol intensity	net indirect	-0.00125	-0.01273	0.01004
Tiger	Patrol intensity	total	-0.00387	-0.02128	0.01277
Leopard	Road pressure	direct	-0.00523	-0.01621	0.00365
Leopard	Road pressure	net indirect	0.00177	-0.00408	0.00913
Leopard	Road pressure	total	-0.00326	-0.01619	0.00834
Leopard	Farmland pressure	direct	-0.00156	-0.01093	0.01181
Leopard	Farmland pressure	net indirect	0.00137	-0.00947	0.01181
Leopard	Farmland pressure	total	0.00013	-0.00991	0.01052
Leopard	Settlement pressure	direct	0.00189	-0.00496	0.00968
Leopard	Settlement pressure	net indirect	0.00138	-0.00289	0.00679
Leopard	Settlement pressure	total	0.00312	-0.00476	0.01210
Leopard	Livestock activity	direct	-0.00098	-0.00631	0.00372
Leopard	Livestock activity	net indirect	0.00049	-0.00254	0.00412
Leopard	Livestock activity	total	-0.00051	-0.00663	0.00520
Leopard	Snaring	direct	-0.00410	-0.01177	0.00231
Leopard	Snaring	net indirect	0.00113	-0.00281	0.00609
Leopard	Snaring	total	-0.00297	-0.01190	0.00526
Leopard	Patrol intensity	direct	-0.00293	-0.01094	0.00357
Leopard	Patrol intensity	net indirect	0.00192	-0.00246	0.00738
Leopard	Patrol intensity	total	-0.00081	-0.00908	0.00663
Tiger	Road pressure	Via Sika deer	0.00637	-0.00071	0.01773
Tiger	Road pressure	Via Roe deer	-0.00807	-0.02105	-0.00025
Tiger	Road pressure	Via Wild boar	-0.00039	-0.00900	0.00266
Tiger	Farmland pressure	Via Sika deer	-0.00133	-0.01004	0.00616
Tiger	Farmland pressure	Via Roe deer	-0.01174	-0.02685	-0.00230
Tiger	Farmland pressure	Via Wild boar	-0.00835	-0.02619	0.00148
Tiger	Settlement pressure	Via Sika deer	0.00136	-0.00509	0.00860
Tiger	Settlement pressure	Via Roe deer	-0.00374	-0.01294	0.00229
Tiger	Settlement pressure	Via Wild boar	-0.00037	-0.00718	0.00199
Tiger	Livestock activity	Via Sika deer	0.00054	-0.00402	0.00562
Tiger	Livestock activity	Via Roe deer	-0.00134	-0.00716	0.00342
Tiger	Livestock activity	Via Wild boar	0.00005	-0.00195	0.00365
Tiger	Snaring	Via Sika deer	-0.00255	-0.00953	0.00238
Tiger	Snaring	Via Roe deer	-0.00300	-0.01062	0.00269
Tiger	Snaring	Via Wild boar	-0.00176	-0.00820	0.00037
Tiger	Patrol intensity	Via Sika deer	-0.00367	-0.01181	0.00216
Tiger	Patrol intensity	Via Roe deer	0.00015	-0.00709	0.00699
Tiger	Patrol intensity	Via Wild boar	0.00234	-0.00049	0.00987
Leopard	Road pressure	Via Sika deer	-0.00141	-0.00493	0.00036
Leopard	Road pressure	Via Roe deer	0.00378	-0.00180	0.01155
Leopard	Road pressure	Via Wild boar	-0.00022	-0.00283	0.00165
Leopard	Farmland pressure	Via Sika deer	0.00026	-0.00160	0.00260
Leopard	Farmland pressure	Via Roe deer	0.00548	-0.00222	0.01503
Leopard	Farmland pressure	Via Wild boar	-0.00449	-0.01210	0.00098
Leopard	Settlement pressure	Via Sika deer	-0.00026	-0.00235	0.00123
Leopard	Settlement pressure	Via Roe deer	0.00172	-0.00159	0.00706
Leopard	Settlement pressure	Via Wild boar	-0.00007	-0.00198	0.00183
Leopard	Livestock activity	Via Sika deer	-0.00011	-0.00145	0.00103
Leopard	Livestock activity	Via Roe deer	0.00062	-0.00191	0.00393
Leopard	Livestock activity	Via Wild boar	0.00000	-0.00132	0.00121
Leopard	Snaring	Via Sika deer	0.00055	-0.00063	0.00251
Leopard	Snaring	Via Roe deer	0.00134	-0.00184	0.00582

Predator	Human variable	Quantity	Median	Lower	Upper
Leopard	Snaring	Via Wild boar	-0.00084	-0.00307	0.00051
Leopard	Patrol intensity	Via Sika deer	0.00079	-0.00054	0.00328
Leopard	Patrol intensity	Via Roe deer	-0.00006	-0.00413	0.00356
Leopard	Patrol intensity	Via Wild boar	0.00116	-0.00058	0.00403

Every candidate product is retained, including products with unsupported components. Product interval exclusion alone does not establish a supported chain. Source data provide component and product screening separately.

Table S3c Snow-free full component coefficients

Points are medians across the 500 original conditional estimates. Lower and upper bounds are the 2.5th and 97.5th percentiles of 200,000 state-and-coefficient simulations. Direction is the dominant sign fraction, not a posterior model probability. Y denotes the descriptive dual support rule. Natural controls are included for completeness.

Response	Predictor	Median	Lower	Upper	Direction	Rule
Sika deer	Road pressure	-0.00135	-0.01182	0.00921	0.5937	–
Sika deer	Farmland pressure	0.00309	-0.00735	0.01349	0.7190	–
Sika deer	Settlement pressure	0.00597	-0.00281	0.01472	0.9078	–
Sika deer	Livestock activity	-0.00232	-0.01039	0.00583	0.7154	–
Sika deer	Snaring	-0.00308	-0.00940	0.00316	0.8333	–
Sika deer	Patrol intensity	-0.00328	-0.01431	0.00759	0.7260	–
Sika deer	Elevation	-0.90847	-0.94769	-0.86931	1.0000	Y
Sika deer	Mean DBH	0.59064	0.54708	0.63390	1.0000	Y
Sika deer	Edge distance	-0.05558	-0.09911	-0.01191	0.9947	Y
Sika deer	Water distance	0.02715	-0.03572	0.09410	0.8189	–
Sika deer	NDVI	0.00637	-0.00548	0.01823	0.8536	–
Sika deer	Snow pressure	0.09383	0.05103	0.13544	1.0000	Y
Roe deer	Road pressure	-0.00367	-0.01849	0.01015	0.6854	–
Roe deer	Farmland pressure	0.00185	-0.01323	0.01605	0.6131	–
Roe deer	Settlement pressure	0.00041	-0.01082	0.01128	0.5312	–
Roe deer	Livestock activity	-0.00458	-0.01583	0.00602	0.8012	–
Roe deer	Snaring	0.00371	-0.00340	0.01126	0.8407	–
Roe deer	Patrol intensity	0.00374	-0.00938	0.01775	0.7042	–
Roe deer	Elevation	0.78559	0.72382	0.84417	1.0000	Y
Roe deer	Mean DBH	0.41880	0.34439	0.49896	1.0000	Y
Roe deer	Edge distance	-0.00034	-0.01176	0.01038	0.5147	–
Roe deer	Water distance	-0.02082	-0.04849	0.00475	0.9436	–
Roe deer	NDVI	-0.08940	-0.12625	-0.04975	1.0000	Y
Roe deer	Snow pressure	0.00953	-0.03255	0.05219	0.6776	–
Wild boar	Road pressure	0.00783	-0.03201	0.04903	0.6514	–
Wild boar	Farmland pressure	0.10831	0.05996	0.16411	1.0000	Y
Wild boar	Settlement pressure	0.00232	-0.03385	0.03808	0.5581	–
Wild boar	Livestock activity	0.01876	-0.01328	0.05244	0.8709	–
Wild boar	Snaring	0.00026	-0.02483	0.02465	0.5173	–
Wild boar	Patrol intensity	-0.01222	-0.05484	0.03047	0.7152	–
Wild boar	Elevation	-0.28460	-0.51340	-0.03084	0.9869	Y
Wild boar	Mean DBH	0.46760	0.20667	0.67620	0.9998	Y
Wild boar	Edge distance	0.00172	-0.03398	0.03820	0.5306	–
Wild boar	Water distance	0.05191	-0.16153	0.27769	0.6752	–
Wild boar	NDVI	-0.20833	-0.28588	-0.13729	1.0000	Y

Response	Predictor	Median	Lower	Upper	Direction	Rule
Wild boar	Snow pressure	-0.12658	-0.26079	0.00035	0.9747	–
Tiger	Sika deer	0.00494	-0.31511	0.31355	0.5159	–
Tiger	Roe deer	-1.08164	-2.03762	-0.52262	1.0000	Y
Tiger	Wild boar	-0.05559	-0.24142	0.04814	0.8314	–
Tiger	Road pressure	-0.00863	-0.03316	0.01617	0.7522	–
Tiger	Farmland pressure	-0.03627	-0.06799	-0.00962	0.9969	Y
Tiger	Settlement pressure	-0.02330	-0.04704	-0.00304	0.9884	Y
Tiger	Livestock activity	0.01868	0.00043	0.03936	0.9778	Y
Tiger	Snaring	-0.00993	-0.02621	0.00431	0.9102	–
Tiger	Patrol intensity	-0.00114	-0.02622	0.02308	0.5424	–
Tiger	Elevation	0.31783	-0.34871	1.17730	0.8215	–
Tiger	Mean DBH	1.08758	0.74749	1.50238	1.0000	Y
Tiger	Edge distance	0.00086	-0.02823	0.02748	0.5147	–
Tiger	Water distance	-0.00868	-0.22824	0.18701	0.5384	–
Tiger	NDVI	0.09773	0.03149	0.18366	0.9986	Y
Tiger	Snow pressure	0.10527	0.00444	0.21863	0.9795	Y
Leopard	Sika deer	-0.03743	-0.12305	0.02547	0.8831	–
Leopard	Roe deer	-0.18131	-0.65917	0.30212	0.7909	–
Leopard	Wild boar	-0.08427	-0.17764	-0.00183	0.9779	Y
Leopard	Road pressure	-0.00385	-0.01791	0.00873	0.7136	–
Leopard	Farmland pressure	0.00943	-0.00209	0.02420	0.9486	–
Leopard	Settlement pressure	0.00181	-0.00644	0.01101	0.6794	–
Leopard	Livestock activity	-0.00889	-0.02012	-0.00038	0.9804	Y
Leopard	Snaring	-0.00049	-0.00634	0.00543	0.5792	–
Leopard	Patrol intensity	-0.00127	-0.01186	0.00889	0.6096	–
Leopard	Elevation	-0.68269	-1.09528	-0.27432	1.0000	Y
Leopard	Mean DBH	1.02969	0.79320	1.22982	1.0000	Y
Leopard	Edge distance	-0.00086	-0.01217	0.00803	0.5741	–
Leopard	Water distance	-0.04131	-0.17800	0.10643	0.7164	–
Leopard	NDVI	-0.00591	-0.03589	0.02910	0.6497	–
Leopard	Snow pressure	-0.02818	-0.07376	0.00940	0.9256	–

Table S3d Snow-free full path decomposition

Predator	Human variable	Quantity	Median	Lower	Upper
Tiger	Road pressure	direct	-0.00863	-0.03316	0.01617
Tiger	Road pressure	net indirect	0.00326	-0.01468	0.01880
Tiger	Road pressure	total	-0.00571	-0.03335	0.02207
Tiger	Farmland pressure	direct	-0.03627	-0.06799	-0.00962
Tiger	Farmland pressure	net indirect	-0.00955	-0.03447	0.00905
Tiger	Farmland pressure	total	-0.04683	-0.08159	-0.01581
Tiger	Settlement pressure	direct	-0.02330	-0.04704	-0.00304
Tiger	Settlement pressure	net indirect	-0.00096	-0.01563	0.01160
Tiger	Settlement pressure	total	-0.02459	-0.05103	-0.00046
Tiger	Livestock activity	direct	0.01868	0.00043	0.03936
Tiger	Livestock activity	net indirect	0.00415	-0.00773	0.01916
Tiger	Livestock activity	total	0.02317	0.00187	0.04621
Tiger	Snaring	direct	-0.00993	-0.02621	0.00431
Tiger	Snaring	net indirect	-0.00433	-0.01367	0.00406
Tiger	Snaring	total	-0.01438	-0.03236	0.00230

Predator	Human variable	Quantity	Median	Lower	Upper
Tiger	Patrol intensity	direct	-0.00114	-0.02622	0.02308
Tiger	Patrol intensity	net indirect	-0.00311	-0.01948	0.01313
Tiger	Patrol intensity	total	-0.00433	-0.03349	0.02469
Leopard	Road pressure	direct	-0.00385	-0.01791	0.00873
Leopard	Road pressure	net indirect	-0.00029	-0.00623	0.00596
Leopard	Road pressure	total	-0.00411	-0.01995	0.01061
Leopard	Farmland pressure	direct	0.00943	-0.00209	0.02420
Leopard	Farmland pressure	net indirect	-0.00986	-0.02050	0.00034
Leopard	Farmland pressure	total	-0.00028	-0.01415	0.01330
Leopard	Settlement pressure	direct	0.00181	-0.00644	0.01101
Leopard	Settlement pressure	net indirect	-0.00042	-0.00540	0.00436
Leopard	Settlement pressure	total	0.00144	-0.00806	0.01152
Leopard	Livestock activity	direct	-0.00889	-0.02012	-0.00038
Leopard	Livestock activity	net indirect	-0.00087	-0.00639	0.00426
Leopard	Livestock activity	total	-0.00994	-0.02281	0.00098
Leopard	Snaring	direct	-0.00049	-0.00634	0.00543
Leopard	Snaring	net indirect	-0.00052	-0.00430	0.00293
Leopard	Snaring	total	-0.00098	-0.00804	0.00596
Leopard	Patrol intensity	direct	-0.00127	-0.01186	0.00889
Leopard	Patrol intensity	net indirect	0.00054	-0.00581	0.00663
Leopard	Patrol intensity	total	-0.00078	-0.01286	0.01140
Tiger	Road pressure	Via Sika deer	-0.00001	-0.00199	0.00176
Tiger	Road pressure	Via Roe deer	0.00380	-0.01407	0.01878
Tiger	Road pressure	Via Wild boar	-0.00044	-0.00505	0.00323
Tiger	Farmland pressure	Via Sika deer	0.00002	-0.00232	0.00188
Tiger	Farmland pressure	Via Roe deer	-0.00206	-0.02436	0.01230
Tiger	Farmland pressure	Via Wild boar	-0.00579	-0.02569	0.00489
Tiger	Settlement pressure	Via Sika deer	0.00003	-0.00272	0.00236
Tiger	Settlement pressure	Via Roe deer	-0.00046	-0.01458	0.01139
Tiger	Settlement pressure	Via Wild boar	-0.00012	-0.00491	0.00266
Tiger	Livestock activity	Via Sika deer	-0.00000	-0.00135	0.00192
Tiger	Livestock activity	Via Roe deer	0.00491	-0.00640	0.01972
Tiger	Livestock activity	Via Wild boar	-0.00102	-0.00558	0.00180
Tiger	Snaring	Via Sika deer	-0.00002	-0.00205	0.00097
Tiger	Snaring	Via Roe deer	-0.00396	-0.01287	0.00404
Tiger	Snaring	Via Wild boar	-0.00004	-0.00337	0.00185
Tiger	Patrol intensity	Via Sika deer	-0.00001	-0.00201	0.00240
Tiger	Patrol intensity	Via Roe deer	-0.00397	-0.01978	0.01148
Tiger	Patrol intensity	Via Wild boar	0.00067	-0.00254	0.00664
Leopard	Road pressure	Via Sika deer	0.00004	-0.00051	0.00078
Leopard	Road pressure	Via Roe deer	0.00031	-0.00396	0.00549
Leopard	Road pressure	Via Wild boar	-0.00065	-0.00507	0.00305
Leopard	Farmland pressure	Via Sika deer	-0.00011	-0.00085	0.00044
Leopard	Farmland pressure	Via Roe deer	-0.00014	-0.00589	0.00385
Leopard	Farmland pressure	Via Wild boar	-0.00937	-0.01933	-0.00020
Leopard	Settlement pressure	Via Sika deer	-0.00022	-0.00104	0.00025
Leopard	Settlement pressure	Via Roe deer	-0.00002	-0.00360	0.00346
Leopard	Settlement pressure	Via Wild boar	-0.00014	-0.00378	0.00339
Leopard	Livestock activity	Via Sika deer	0.00007	-0.00037	0.00063
Leopard	Livestock activity	Via Roe deer	0.00061	-0.00294	0.00473
Leopard	Livestock activity	Via Wild boar	-0.00154	-0.00575	0.00115
Leopard	Snaring	Via Sika deer	0.00011	-0.00013	0.00077

Predator	Human variable	Quantity	Median	Lower	Upper
Leopard	Snaring	Via Roe deer	-0.00066	-0.00392	0.00154
Leopard	Snaring	Via Wild boar	-0.00001	-0.00243	0.00251
Leopard	Patrol intensity	Via Sika deer	0.00011	-0.00046	0.00090
Leopard	Patrol intensity	Via Roe deer	-0.00059	-0.00584	0.00322
Leopard	Patrol intensity	Via Wild boar	0.00102	-0.00287	0.00577

Every candidate product is retained, including products with unsupported components. Product interval exclusion alone does not establish a supported chain. Source data provide component and product screening separately.

Table S4 Detailed independence-test summaries

Season	Claim pair	Median beta	Median z	Median log10 p	Reject percent
Snow-free	Sika deer / boar	0.00737	1.32928	-0.82102	33.4
Snow-free	Sika deer / roe deer	-0.01672	-0.89743	-0.439	3.2
Snow-free	Roe deer / boar	0.0246	3.4896	-3.48294	74.6
Snow-free	Tiger / leopard	-0.63168	-11.12509	-29.19979	93.2
Snow	Sika deer / boar	-0.15616	-10.53037	-27.69424	91.8
Snow	Sika deer / roe deer	0.3298	12.96272	-37.701	100.0
Snow	Roe deer / boar	-0.04795	-3.2397	-3.23595	74.4
Snow	Tiger / leopard	0.4248	8.3883	-16.5649	91.6

Prey-pair tests condition on all six human variables and six natural controls. Predator-pair tests additionally condition on all three prey. Every row summarizes 500 spatial-error regressions at $n = 1,824$. Rejection percentages use within-fit two-sided Normal coefficient tests at 0.05; they are not posterior probabilities.

Table S5a Fisher diagnostics

Season	Draws	Median C	2.5 percent	97.5 percent	Pass percent
Snow-free	500	158.06164	14.68425	1263.05033	3.0
Snow	500	497.77655	164.25669	1593.87201	0.0

Each main-model Fisher test has four claims and eight degrees of freedom. The interval summarizes Fisher C across state draws, not a confidence interval for model fit.

Table S5b Spatial error parameters

Season	Equation	Median	2.5 percent	97.5 percent
Snow-free	deer _||_ boar | exogenous	0.908	0.898	0.9183
Snow-free	deer _||_ roe | exogenous	0.90909	0.90033	0.91871
Snow-free	roe _||_ boar | exogenous	0.86772	0.85799	0.8811
Snow-free	tiger _||_ leopard | prey + exogenous	0.88231	0.85026	0.90629
Snow-free	boar structural equation	0.8177	0.79206	0.84794
Snow-free	deer structural equation	0.90887	0.9001	0.9185
Snow-free	leopard structural equation	0.85498	0.83445	0.88067
Snow-free	roe structural equation	0.86512	0.85588	0.87914

Season	Equation	Median	2.5 percent	97.5 percent
Snow-free	tiger structural equation	0.88786	0.85696	0.90936
Snow	deer _||_ boar | exogenous	0.93878	0.90987	0.95903
Snow	deer _||_ roe | exogenous	0.92219	0.89873	0.94713
Snow	roe _||_ boar | exogenous	0.88232	0.86981	0.90372
Snow	tiger _||_ leopard | prey + exogenous	0.88236	0.83294	0.91038
Snow	boar structural equation	0.8443	0.79354	0.92386
Snow	deer structural equation	0.92711	0.90588	0.95058
Snow	leopard structural equation	0.84887	0.75157	0.92643
Snow	roe structural equation	0.879	0.86813	0.89865
Snow	tiger structural equation	0.888	0.85531	0.91119

Table S5c Filtered innovation Moran diagnostics

Season	Equation	Median	2.5 percent	97.5 percent
Snow-free	deer _||_ boar | exogenous	-0.03901	-0.04078	-0.03661
Snow-free	deer _||_ roe | exogenous	-0.03941	-0.04074	-0.03733
Snow-free	roe _||_ boar | exogenous	-0.02062	-0.02932	-0.00674
Snow-free	tiger _||_ leopard | prey + exogenous	-0.02156	-0.03569	-0.00838
Snow-free	boar structural equation	-0.0327	-0.03986	-0.02053
Snow-free	deer structural equation	-0.03972	-0.04092	-0.03775
Snow-free	leopard structural equation	-0.04234	-0.05193	-0.01972
Snow-free	roe structural equation	-0.01868	-0.02934	-0.00404
Snow-free	tiger structural equation	-0.01682	-0.02768	-0.00676
Snow	deer _||_ boar | exogenous	-0.02742	-0.04029	-0.00952
Snow	deer _||_ roe | exogenous	-0.02887	-0.03791	-0.02229
Snow	roe _||_ boar | exogenous	-0.01412	-0.02704	0.00022
Snow	tiger _||_ leopard | prey + exogenous	-0.01733	-0.04026	-0.0007
Snow	boar structural equation	-0.03434	-0.04642	-0.01414
Snow	deer structural equation	-0.02354	-0.031	-0.01791
Snow	leopard structural equation	-0.03992	-0.06076	-0.01507
Snow	roe structural equation	-0.01281	-0.02555	0.00105
Snow	tiger structural equation	-0.0151	-0.03047	-0.00229

Table S5d Definitions of six park-wide alternatives

A1–A4 use ordinary linear regressions; M1, A5 and A6 use the spatial-error framework. All models use the same 1,824-grid support and separate seasonal analyses. State variables are transformed and standardized within their respective fitting pipelines. The original B–H–M classification and scenario models are not replaced by these park-wide alternatives.

Model	Specification	Comparison target and change	Interpretation boundary
M1	Primary spatial-error	Six human variables, six natural controls, three simultaneous prey, 500 state pairs	Four independence claims; no predator arrow or residual association.
A1	Ordinary LM	M1 without spatial-error regressions; 500 state pairs	Different archived pairing seed from M1. Differences are not paired spatial-correction effects.

Model	Specification	Comparison target and change	Interpretation boundary
A2	Posterior-mean LM	A1 using posterior means; one fit per season	No state-propagation interval. Ordinary regression standard errors do not replace state uncertainty.
A3	Composite-HDI LM	A1 replacing six separate human predictors with one saved HDI predictor	Changes the exposure estimand. HDI composition and weights require author verification.
A4	No-natural-controls LM	A1 omitting the six natural controls	Assesses adjustment sensitivity within the LM family; unadjusted associations may reflect confounding.
A5	Tiger/leopard residual association	M1 structural coefficients retained; predator-pair independence claim removed	Seasonal innovation-residual correlation is descriptive, not directed. No new structural coefficients are fitted.
A6	Tiger to leopard	M1 with tiger state added to the leopard equation; predator independence claim removed	Refitted leopard equation and extended indirect paths do not identify competition or causal direction.

A1, A3 and A4 share their archived draw indices. M1 and A6 also share their archived draw indices, but none of the 500 same-index pairs match between the ordinary-LM and spatial families. A2–A4 therefore use A1 as their immediate comparison. A5 is derived from M1 output and is not counted as 500 new structural fits. These models address several forms of sensitivity rather than one likelihood-based candidate ranking. No AIC ranking across changed exposure sets, residual-claim sets or input-state definitions is inferred.

Table S5e Fit diagnostics for the primary model and alternatives

Model	Season	State sets	Claims	Median C	$p > 0.05$ percent
M1	Snow	500	4	497.78	0.0
M1	Snow-free	500	4	158.06	3.0
A1	Snow	500	4	503.59	0.0
A1	Snow-free	500	4	175.18	0.6
A2	Snow	1	4	538.53	0.0
A2	Snow-free	1	4	199.54	0.0
A3	Snow	500	4	468.14	0.0
A3	Snow-free	500	4	205.89	0.8
A4	Snow	500	4	4215.45	0.0
A4	Snow-free	500	4	2128.35	0.0
A5	Snow	500	3	347.03	0.0
A5	Snow-free	500	3	25.20	22.4
A6	Snow	500	3	347.03	0.0
A6	Snow-free	500	3	25.20	22.4

Fisher's C has twice the number of retained claims as degrees of freedom. Passing fractions summarize within-fit diagnostics, not posterior model probabilities, biological replicates or formal evidence for one graph over another. A2 has one posterior-mean fit per season, so its percentage describes one diagnostic outcome and is not comparable to a 500-draw uncertainty distribution. All independent archived structural fits succeeded; successful computation is distinct from adequate network fit. A5 and A6 retain the same three prey-pair tests, giving identical Fisher diagnostics even though their predator equations differ.

Table S5f Full alternative coefficients and quantities

The accompanying `Table_S5f_all_component_coefficients.csv` reports all archived component coefficient summaries with their exact response and predictor labels, including unsupported estimates. A5

structural summaries duplicate M1 by construction. A2 intervals are left missing because only a posterior-mean state fit is used; ordinary-LM standard errors remain in the original archive and are not relabeled as state intervals. Model-specific R-squared and LM effect summaries accompany the tables. `Table_S5f_spatial_effect_summary.csv` retains the primary effects and the extended A6 paths through tiger, with A5 effects inherited from M1 by construction. Quantities under a composite HDI or a tiger-to-leopard arrow address different conditional paths and are not substituted for the six-disturbance main effects.

Table S5g Predator-path sensitivity

Model	Season	Predictor / outcome	Median	2.5 percent	97.5 percent
A1	Snow-free	Wild boar / Leopard	-0.0687	-0.1436	0.0024
A1	Snow-free	Sika deer / Leopard	-0.1081	-0.2158	-0.0167
A1	Snow-free	Roe deer / Leopard	-0.1442	-0.5993	0.2941
A1	Snow-free	Wild boar / Tiger	-0.1070	-0.3275	0.0396
A1	Snow-free	Sika deer / Tiger	0.3287	-0.0204	0.5383
A1	Snow-free	Roe deer / Tiger	-1.2700	-2.2980	-0.6386
A1	Snow	Wild boar / Leopard	-0.0389	-0.2066	0.0970
A1	Snow	Sika deer / Leopard	-0.0982	-0.2613	0.0377
A1	Snow	Roe deer / Leopard	0.2878	-0.0821	0.6935
A1	Snow	Wild boar / Tiger	-0.1005	-0.5722	0.1371
A1	Snow	Sika deer / Tiger	0.4950	0.3028	0.8054
A1	Snow	Roe deer / Tiger	-0.5813	-1.3299	-0.1673
A2	Snow-free	Wild boar / Leopard	-0.0646	–	–
A2	Snow-free	Sika deer / Leopard	-0.1087	–	–
A2	Snow-free	Roe deer / Leopard	-0.1804	–	–
A2	Snow-free	Wild boar / Tiger	-0.1084	–	–
A2	Snow-free	Sika deer / Tiger	0.3501	–	–
A2	Snow-free	Roe deer / Tiger	-1.3353	–	–
A2	Snow	Wild boar / Leopard	-0.0398	–	–
A2	Snow	Sika deer / Leopard	-0.1164	–	–
A2	Snow	Roe deer / Leopard	0.3397	–	–
A2	Snow	Wild boar / Tiger	-0.1028	–	–
A2	Snow	Sika deer / Tiger	0.5589	–	–
A2	Snow	Roe deer / Tiger	-0.6479	–	–
A3	Snow-free	Wild boar / Leopard	-0.0588	-0.1264	0.0011
A3	Snow-free	Sika deer / Leopard	-0.1236	-0.2490	-0.0215
A3	Snow-free	Roe deer / Leopard	-0.1885	-0.6508	0.2473
A3	Snow-free	Wild boar / Tiger	-0.1020	-0.3271	0.0428
A3	Snow-free	Sika deer / Tiger	0.3666	0.0096	0.5930
A3	Snow-free	Roe deer / Tiger	-1.2701	-2.3068	-0.6579
A3	Snow	Wild boar / Leopard	-0.0295	-0.1759	0.0995
A3	Snow	Sika deer / Leopard	-0.0995	-0.2654	0.0338
A3	Snow	Roe deer / Leopard	0.2875	-0.0871	0.7008
A3	Snow	Wild boar / Tiger	-0.1134	-0.5792	0.1249
A3	Snow	Sika deer / Tiger	0.4896	0.2988	0.7941
A3	Snow	Roe deer / Tiger	-0.5566	-1.2977	-0.1509
A4	Snow-free	Wild boar / Leopard	0.0377	-0.0508	0.1771
A4	Snow-free	Sika deer / Leopard	0.9904	0.8539	1.0789
A4	Snow-free	Roe deer / Leopard	0.2806	0.1282	0.4595
A4	Snow-free	Wild boar / Tiger	-0.0165	-0.2030	0.1512
A4	Snow-free	Sika deer / Tiger	0.7016	0.4576	0.9429
A4	Snow-free	Roe deer / Tiger	0.1012	-0.1718	0.3801
A4	Snow	Wild boar / Leopard	-0.4744	-0.8901	-0.0799
A4	Snow	Sika deer / Leopard	0.3270	0.0203	0.6988
A4	Snow	Roe deer / Leopard	-0.7254	-1.0639	-0.3049
A4	Snow	Wild boar / Tiger	-0.4460	-0.8128	-0.0948
A4	Snow	Sika deer / Tiger	0.2954	0.0139	0.6482
A4	Snow	Roe deer / Tiger	-0.7627	-1.0540	-0.4459

Model	Season	Predictor / outcome	Median	2.5 percent	97.5 percent
A5	Snow-free	Wild boar / Leopard	-0.0843	-0.1754	-0.0033
A5	Snow-free	Sika deer / Leopard	-0.0374	-0.1051	0.0143
A5	Snow-free	Roe deer / Leopard	-0.1813	-0.6613	0.2885
A5	Snow-free	Wild boar / Tiger	-0.0556	-0.2339	0.0461
A5	Snow-free	Sika deer / Tiger	0.0049	-0.2968	0.3039
A5	Snow-free	Roe deer / Tiger	-1.0816	-2.0283	-0.5162
A5	Snow	Wild boar / Leopard	-0.0649	-0.2295	0.0344
A5	Snow	Sika deer / Leopard	-0.1027	-0.2415	0.0033
A5	Snow	Roe deer / Leopard	0.2683	-0.0848	0.6975
A5	Snow	Wild boar / Tiger	-0.1316	-0.6797	0.0167
A5	Snow	Sika deer / Tiger	0.4775	0.2051	0.8377
A5	Snow	Roe deer / Tiger	-0.5546	-1.2877	-0.1774
A6	Snow-free	Wild boar / Leopard	-0.0896	-0.1800	-0.0032
A6	Snow-free	Sika deer / Leopard	-0.0373	-0.1010	0.0157
A6	Snow-free	Roe deer / Leopard	-0.2947	-0.9953	0.3637
A6	Snow-free	Tiger / Leopard	-0.0868	-0.2905	0.0797
A6	Snow-free	Wild boar / Tiger	-0.0556	-0.2339	0.0461
A6	Snow-free	Sika deer / Tiger	0.0049	-0.2968	0.3039
A6	Snow-free	Roe deer / Tiger	-1.0816	-2.0283	-0.5162
A6	Snow	Wild boar / Leopard	-0.0514	-0.2080	0.0740
A6	Snow	Sika deer / Leopard	-0.1505	-0.3518	0.0022
A6	Snow	Roe deer / Leopard	0.3322	-0.1014	0.8258
A6	Snow	Tiger / Leopard	0.0831	-0.0331	0.3099
A6	Snow	Wild boar / Tiger	-0.1316	-0.6797	0.0167
A6	Snow	Sika deer / Tiger	0.4775	0.2051	0.8377
A6	Snow	Roe deer / Tiger	-0.5546	-1.2877	-0.1774
M1	Snow-free	Wild boar / Leopard	-0.0843	-0.1754	-0.0033
M1	Snow-free	Sika deer / Leopard	-0.0374	-0.1051	0.0143
M1	Snow-free	Roe deer / Leopard	-0.1813	-0.6613	0.2885
M1	Snow-free	Wild boar / Tiger	-0.0556	-0.2339	0.0461
M1	Snow-free	Sika deer / Tiger	0.0049	-0.2968	0.3039
M1	Snow-free	Roe deer / Tiger	-1.0816	-2.0283	-0.5162
M1	Snow	Wild boar / Leopard	-0.0649	-0.2295	0.0344
M1	Snow	Sika deer / Leopard	-0.1027	-0.2415	0.0033
M1	Snow	Roe deer / Leopard	0.2683	-0.0848	0.6975
M1	Snow	Wild boar / Tiger	-0.1316	-0.6797	0.0167
M1	Snow	Sika deer / Tiger	0.4775	0.2051	0.8377
M1	Snow	Roe deer / Tiger	-0.5546	-1.2877	-0.1774

These are conditional standardized coefficients at the respective state realizations, not causal effects. Intervals summarize propagated states and omit regression-parameter and imputation uncertainty. Signs, interval inclusion and magnitudes must be evaluated together. A5 residual correlations are in `A5_residual_correlation_summary.csv`. Their intervals cross zero in both seasons, which does not prove an absence of ecological interaction.

Note S6 Scope of the retained B–H–M analysis

The original classification, scenario models and numerical pathway outputs are retained. Main Figures 12–15 present the four complete original scenario images. Their archived direction symbols omit within-fit coefficient uncertainty and are not directly comparable with the extended park-wide intervals. The additions below describe classification coverage and cutoff sensitivity, not new SEM fits.

Table S6a Eight-context classification inventory

Across all 1,824 archived grids, the eight focal contexts contain 493 grids (27.03%) and the background class contains 1,331 (72.97%). The individual context counts, in Table S6a order, are 127, 118, 11, 8, 50, 110, 38 and 31. Classified grids and grids actually retained by a fitted SEM are distinct quantities.

Context	Classified n	Percent	Path diagram	Fitted sample documentation
B high H low M high	127	6.96	Reported	Effective n unverified
B high H high M high	118	6.47	Reported	Effective n unverified
B high H low M low	11	0.60	Not reported	Selection record unavailable
B high H high M low	8	0.44	Not reported	Selection record unavailable
B low H low M low	50	2.74	Not reported	Selection record unavailable
B low H high M low	110	6.03	Reported	Effective n unverified
B low H low M high	38	2.08	Reported	Effective n unverified
B low H high M high	31	1.70	Not reported	Selection record unavailable
Background	1331	72.97	Not reported	Not a focal comparison
Total	1,824	100.00		

The inventory counts entire analytical cells, including boundary cells, without an additional map-display or area filter. High M denotes core protection zoning and low M denotes general control zoning. The 11- and 8-grid high-B general-control classes are small, but counts alone do not establish why the 31- and 50-grid classes were not reported, or the estimability of any particular equation. A model-specific sample list and the recorded selection basis are still required. No minimum sample-size rule is reconstructed retrospectively.

Table S6b Classification-only cutoff comparison

Changing the symmetric cutoffs yields 334 focal grids at the 20th/80th percentiles, 493 at the retained 25th/75th percentiles, and 677 at the 30th/70th percentiles. The corresponding background counts are 1,490, 1,331 and 1,147. All 334 stricter-cutoff focal grids retain their baseline class; all 493 baseline focal grids retain their class under the broader cutoffs. The high-B–high-H–low-M context changes from 1 to 8 to 25 grids. Thus class sizes depend materially on the cutoff, even though all eight class labels remain represented. This check does not establish stable pathway signs, interval support or management rankings under alternative thresholds.

Context	20th/80th	25th/75th	30th/70th
B+ H- M+	93	127	159
B+ H+ M+	77	118	147
B+ H- M-	5	11	26
B+ H+ M-	1	8	25
B- H- M-	25	50	68
B- H+ M-	85	110	149
B- H- M+	28	38	60
B- H+ M+	20	31	43
Background	1490	1331	1147
Focal subtotal	334	493	677
Total	1,824	1,824	1,824

Methods S6c Inventory and cutoff calculations

The supplied processed classification contains 1,824 unique grid IDs, continuous B and H scores, binary M and saved class labels. Linear interpolated empirical quantiles (R type 7 / pandas linear) were calculated over the same full set of grids. A score strictly below its lower cutoff was low, a score strictly above its upper cutoff was high, and intermediate scores were background. The 25th/75th calculation reproduces all 1,824 saved class labels exactly. M, the primary class labels and every archived SEM remain unchanged. Alternative-cutoff

classes are separate descriptive comparisons. The source tables contain cutoffs, per-class counts and grid-level membership. No model fit, coefficient, uncertainty interval or path-support statistic was recomputed.

Note S6d Ecological meaning of a common spatial support

The spatial unit is a 3-km by 3-km landscape patch, not an individual territory or an encounter radius. Wang et al. (2018, doi:10.1016/j.biocon.2017.11.008) interpret local Amur tiger camera detections below home-range scale as habitat use. Rozhnov et al. (2015, doi:10.1134/S1062359015090095) followed one female Amur leopard with GPS telemetry and documented reproductive-state variation in home-range and core-area use. This case does not define a population-wide home-range size. Feng et al. (2021, doi:10.1007/s10980-021-01226-6) report camera-site-scale livestock associations with sika deer, Siberian roe deer and wild boar. These sources support distinguishing predator movement from local prey use, not a claim that 9 square kilometres is biologically optimal for every species.

Using one lattice gives the linked regressions a common spatial reference while retaining species-specific ecological and observation models. The existing 1-, 3- and 5-km IDM comparison addresses detection information, model stability and spatial resolution. It does not test SEM pathway directions across scales, and its fitting-data AUC is not independent predictive validation. The present inference is therefore explicitly conditional on the 3-km support. Species-specific movement data and comparable alternative-scale pathway fits remain future checks.

Table S7 Four working hypotheses and all comparisons

H1 predicts positive prey–predator associations; H2 negative pressure–prey associations; H3 negative direct pressure–predator associations; H4 negative prey-mediated associations with the component sequence predicted by H1 and H2. The full pre-screening network contains, per season, 12 direct human-variable–predator paths, 18 human-variable–prey paths, six prey–predator paths, and 36 possible single-prey chains; controls are excluded from these counts. H1–H4 cover the five disturbance pressures, while patrol-intensity paths are evaluated separately. H4 is derived from component expectations rather than an independent mechanism test. Net sums are shown below and every prey-specific product is in Table S3. Nominal intervals and exploratory screening are not multiplicity-adjusted confirmatory tests. The framework is articulated during revision unless earlier dated documentation is supplied.

H	Season	Predictor / response	Median	Lower	Upper	Evaluation
H1	Snow-free	Wild boar / Leopard	-0.08427	-0.17764	-0.00183	Opposite direction
H1	Snow-free	Wild boar / Tiger	-0.05559	-0.24142	0.04814	Unresolved
H1	Snow-free	Sika deer / Leopard	-0.03743	-0.12305	0.02547	Unresolved
H1	Snow-free	Sika deer / Tiger	0.00494	-0.31511	0.31355	Unresolved
H1	Snow-free	Roe deer / Leopard	-0.18131	-0.65917	0.30212	Unresolved
H1	Snow-free	Roe deer / Tiger	-1.08164	-2.03762	-0.52262	Opposite direction
H1	Snow	Wild boar / Leopard	-0.06488	-0.23063	0.03760	Unresolved
H1	Snow	Wild boar / Tiger	-0.13161	-0.67490	0.02557	Unresolved
H1	Snow	Sika deer / Leopard	-0.10265	-0.24480	0.00610	Unresolved
H1	Snow	Sika deer / Tiger	0.47747	0.19240	0.85227	Expected direction
H1	Snow	Roe deer / Leopard	0.26832	-0.09535	0.69628	Unresolved
H1	Snow	Roe deer / Tiger	-0.55458	-1.29236	-0.16861	Opposite direction
H2	Snow-free	Farmland pressure / Wild boar	0.10831	0.05996	0.16411	Opposite direction
H2	Snow-free	Farmland pressure / Sika deer	0.00309	-0.00735	0.01349	Unresolved

H	Season	Predictor / response	Median	Lower	Upper	Evaluation
H2	Snow-free	Farmland pressure / Roe deer	0.00185	-0.01323	0.01605	Unresolved
H2	Snow-free	Livestock activity / Wild boar	0.01876	-0.01328	0.05244	Unresolved
H2	Snow-free	Livestock activity / Sika deer	-0.00232	-0.01039	0.00583	Unresolved
H2	Snow-free	Livestock activity / Roe deer	-0.00458	-0.01583	0.00602	Unresolved
H2	Snow-free	Road pressure / Wild boar	0.00783	-0.03201	0.04903	Unresolved
H2	Snow-free	Road pressure / Sika deer	-0.00135	-0.01182	0.00921	Unresolved
H2	Snow-free	Road pressure / Roe deer	-0.00367	-0.01849	0.01015	Unresolved
H2	Snow-free	Snaring / Wild boar	0.00026	-0.02483	0.02465	Unresolved
H2	Snow-free	Snaring / Sika deer	-0.00308	-0.00940	0.00316	Unresolved
H2	Snow-free	Snaring / Roe deer	0.00371	-0.00340	0.01126	Unresolved
H2	Snow-free	Settlement pressure / Wild boar	0.00232	-0.03385	0.03808	Unresolved
H2	Snow-free	Settlement pressure / Sika deer	0.00597	-0.00281	0.01472	Unresolved
H2	Snow-free	Settlement pressure / Roe deer	0.00041	-0.01082	0.01128	Unresolved
H2	Snow	Farmland pressure / Wild boar	0.06650	0.00899	0.12267	Opposite direction
H2	Snow	Farmland pressure / Sika deer	-0.00303	-0.01898	0.01180	Unresolved
H2	Snow	Farmland pressure / Roe deer	0.02126	0.00637	0.03822	Opposite direction
H2	Snow	Livestock activity / Wild boar	-0.00022	-0.01442	0.01491	Unresolved
H2	Snow	Livestock activity / Sika deer	0.00119	-0.00774	0.01054	Unresolved
H2	Snow	Livestock activity / Roe deer	0.00250	-0.00553	0.01089	Unresolved
H2	Snow	Road pressure / Wild boar	0.00438	-0.02002	0.02555	Unresolved
H2	Snow	Road pressure / Sika deer	0.01427	-0.00165	0.02985	Unresolved
H2	Snow	Road pressure / Roe deer	0.01489	0.00076	0.03003	Opposite direction
H2	Snow	Snaring / Wild boar	0.01249	-0.00281	0.03198	Unresolved
H2	Snow	Snaring / Sika deer	-0.00565	-0.01728	0.00485	Unresolved
H2	Snow	Snaring / Roe deer	0.00575	-0.00417	0.01667	Unresolved
H2	Snow	Settlement pressure / Wild boar	0.00239	-0.02036	0.02332	Unresolved
H2	Snow	Settlement pressure / Sika deer	0.00290	-0.01011	0.01556	Unresolved
H2	Snow	Settlement pressure / Roe deer	0.00661	-0.00431	0.01820	Unresolved
H3	Snow-free	Farmland pressure / Leopard	0.00943	-0.00209	0.02420	Unresolved
H3	Snow-free	Farmland pressure / Tiger	-0.03627	-0.06799	-0.00962	Expected direction
H3	Snow-free	Livestock activity / Leopard	-0.00889	-0.02012	-0.00038	Expected direction
H3	Snow-free	Livestock activity / Tiger	0.01868	0.00043	0.03936	Opposite direction
H3	Snow-free	Road pressure / Leopard	-0.00385	-0.01791	0.00873	Unresolved
H3	Snow-free	Road pressure / Tiger	-0.00863	-0.03316	0.01617	Unresolved
H3	Snow-free	Snaring / Leopard	-0.00049	-0.00634	0.00543	Unresolved
H3	Snow-free	Snaring / Tiger	-0.00993	-0.02621	0.00431	Unresolved

H	Season	Predictor / response	Median	Lower	Upper	Evaluation
H3	Snow-free	Settlement pressure / Leopard	0.00181	-0.00644	0.01101	Unresolved
H3	Snow-free	Settlement pressure / Tiger	-0.02330	-0.04704	-0.00304	Expected direction
H3	Snow	Farmland pressure / Leopard	-0.00156	-0.01093	0.01181	Unresolved
H3	Snow	Farmland pressure / Tiger	-0.00858	-0.03282	0.01041	Unresolved
H3	Snow	Livestock activity / Leopard	-0.00098	-0.00631	0.00372	Unresolved
H3	Snow	Livestock activity / Tiger	0.00183	-0.00843	0.01233	Unresolved
H3	Snow	Road pressure / Leopard	-0.00523	-0.01621	0.00365	Unresolved
H3	Snow	Road pressure / Tiger	-0.00122	-0.01964	0.01692	Unresolved
H3	Snow	Snaring / Leopard	-0.00410	-0.01177	0.00231	Unresolved
H3	Snow	Snaring / Tiger	0.00232	-0.01014	0.01508	Unresolved
H3	Snow	Settlement pressure / Leopard	0.00189	-0.00496	0.00968	Unresolved
H3	Snow	Settlement pressure / Tiger	-0.01254	-0.02975	0.00187	Unresolved
H4	Snow-free	Farmland pressure / Leopard	-0.00986	-0.02050	0.00034	Unresolved
H4	Snow-free	Farmland pressure / Tiger	-0.00955	-0.03447	0.00905	Unresolved
H4	Snow-free	Livestock activity / Leopard	-0.00087	-0.00639	0.00426	Unresolved
H4	Snow-free	Livestock activity / Tiger	0.00415	-0.00773	0.01916	Unresolved
H4	Snow-free	Road pressure / Leopard	-0.00029	-0.00623	0.00596	Unresolved
H4	Snow-free	Road pressure / Tiger	0.00326	-0.01468	0.01880	Unresolved
H4	Snow-free	Snaring / Leopard	-0.00052	-0.00430	0.00293	Unresolved
H4	Snow-free	Snaring / Tiger	-0.00433	-0.01367	0.00406	Unresolved
H4	Snow-free	Settlement pressure / Leopard	-0.00042	-0.00540	0.00436	Unresolved
H4	Snow-free	Settlement pressure / Tiger	-0.00096	-0.01563	0.01160	Unresolved
H4	Snow	Farmland pressure / Leopard	0.00137	-0.00947	0.01181	Unresolved
H4	Snow	Farmland pressure / Tiger	-0.02212	-0.04478	-0.00517	Net direction only
H4	Snow	Livestock activity / Leopard	0.00049	-0.00254	0.00412	Unresolved
H4	Snow	Livestock activity / Tiger	-0.00058	-0.00825	0.00680	Unresolved
H4	Snow	Road pressure / Leopard	0.00177	-0.00408	0.00913	Unresolved
H4	Snow	Road pressure / Tiger	-0.00246	-0.01798	0.01103	Unresolved
H4	Snow	Snaring / Leopard	0.00113	-0.00281	0.00609	Unresolved
H4	Snow	Snaring / Tiger	-0.00787	-0.01883	0.00053	Unresolved
H4	Snow	Settlement pressure / Leopard	0.00138	-0.00289	0.00679	Unresolved
H4	Snow	Settlement pressure / Tiger	-0.00320	-0.01516	0.00675	Unresolved

"Net direction only" indicates a negative interval for the net sum; it does not establish the negative-pressure–prey/positive-prey–predator sequence. No pressure–prey coefficient supported the expected negative direction in either season under the extended rule, so no entire chain substantiated that predicted sequence. A negative net value can arise through opposite component signs, as for farmland–roe deer–tiger in snow.

Methods S8 Conditional coefficient uncertainty and imputation sensitivity

For a component equation, let $A_j = I - \widehat{\rho}_j W$, $X_j^* = A_j X_j$ and $y_j^* = A_j y_j$. Conditional on the fitted spatial parameter,

$$\widehat{\boldsymbol{\beta}}_j = (X_j^{*\top} X_j^*)^{-1} X_j^{*\top} y_j^*, \qquad V_j = \widehat{\sigma}_j^2 (X_j^{*\top} X_j^*)^{-1}, \quad \widehat{\sigma}_j^2 = \|y_j^* - X_j^* \widehat{\boldsymbol{\beta}}_j\|^2 / n.$$

The intercept is retained in covariance calculation and omitted from reported pathways. The transformed-design covariance reproduces the archived asymptotic coefficient standard errors, including within-equation correlations. Recovery uses the same 500 state pairings, permanent grid alignment, exogenous transformations and

spatial parameters; it changes neither the original point fits nor B–H–M models. All 5,000 equation-by-state recoveries pass a 10^{-6} coefficient/standard-error agreement threshold, with positive-definite covariance matrices. The supplied QC table retains every discrepancy. The large sparse-fit objects did not expose a usable full covariance through the installed `vcov.Sarlm` method, so the validated transformed-design expression was used rather than assuming independent coefficients. See the official spatialreg maximum-likelihood model documentation and the archived fitting code.

Within each state pair we simulate 400 coefficient vectors from $N(\widehat{\boldsymbol{\beta}}_j, V_j)$ for each component equation, preserving its coefficient covariance. We recompute every product and all-prey sum within the same simulation. Between-equation coefficient vectors are conditionally independent under the working SEM; a complete cross-equation posterior covariance is not estimated. Spatial parameters, the completed primary covariates, graph choice and B/H classifications are held fixed. The resulting 200,000 simulations per season are computational replicates rather than independent ecological observations. The 2.5th–97.5th percentiles are approximate conditional simulation intervals, not a full Bayesian posterior or an interval over graph selection. The seed is 20261016. Revision-stage calculations use R 4.6.1; the archived fit version remains reported in the main Methods.

A separate sensitivity draws 500 joint GAM coefficient vectors from the archived livestock model using its covariance including the available smoothing-parameter correction (`mgcv` 1.9-4; seed 20261017). Predictions reproduce the archived fitted means within 6×10^{-10} on the modeled scale. Each draw updates only the 1,368 previously filled entries, leaves 2,280 observed entries fixed and standardizes within season. One imputation draw is paired with each original state pair. Conditional SEM coefficients and covariance are recalculated at the corresponding original spatial parameter, then the same coefficient simulation is applied. This is mean-function and coefficient sensitivity, not full multiple imputation: residual prediction error, unknown missingness processes, raw observation error and re-estimation of spatial parameters are outside it. Both seasons share each joint GAM draw; original B–H–M membership is untouched.

The primary and GAM-sensitive analyses retain the same descriptive count pattern: 0 direct/2 single-prey chains in snow and 4 direct/1 chain in snow-free conditions. Full GAM-sensitive coefficients, products and effect intervals are supplied with the prefix `v5_GAM_`. Half-simulation interval comparisons document Monte Carlo variation; no classification is selected by switching simulation seeds. These checks do not remedy poor global network fit.

Table S8 Winter leopard precision

The six prey–predator contrasts below separate precision from pathway absence. They are not formal power estimates. Winter leopard cameras have 310 positive grid-months, and tiger cameras 210. Conditional spatial gradients, covariate correlation and state uncertainty also affect pathway precision.

Predator	Prey	Median	Lower	Upper	Width
Tiger	Sika deer	0.47747	0.19240	0.85227	0.65987
Tiger	Roe deer	-0.55458	-1.29236	-0.16861	1.12375
Tiger	Wild boar	-0.13161	-0.67490	0.02557	0.70046
Leopard	Sika deer	-0.10265	-0.24480	0.00610	0.25091
Leopard	Roe deer	0.26832	-0.09535	0.69628	0.79163
Leopard	Wild boar	-0.06488	-0.23063	0.03760	0.26823
